# PARP1-produced poly-ADP-ribose causes the PARP12 translocation to stress granules and impairment of Golgi complex functions

**DOI:** 10.1038/s41598-017-14156-8

**Published:** 2017-10-25

**Authors:** Giuliana Catara, Giovanna Grimaldi, Laura Schembri, Daniela Spano, Gabriele Turacchio, Matteo Lo Monte, Andrea Rosario Beccari, Carmen Valente, Daniela Corda

**Affiliations:** 1 Institute of Protein Biochemistry, National Research Council, Naples, Via Pietro Castellino 111, 80131 Italy; 2 Dompé Farmaceutici SpA Research Center, L’Aquila, Via Campo di Pile, 67100 Italy

## Abstract

Poly-ADP-ribose-polymerases (PARPs) 1 and 2 are nuclear enzymes that catalyze the poly-ADP-ribosylation of nuclear proteins transferring poly-ADP-ribose (PAR) polymers to specific residues. PARPs and PAR intervene in diverse functions, including DNA repair in the nucleus and stress granule assembly in the cytoplasm. Stress granules contribute to the regulation of translation by clustering and stabilizing mRNAs as well as several cytosolic PARPs and signaling proteins to modulate cell metabolism and survival. Our study is focused on one of these PARPs, PARP12, a Golgi-localized mono-ADP-ribosyltransferase that under stress challenge reversibly translocates from the Golgi complex to stress granules. PARP1 activation and release of nuclear PAR drive this translocation by direct PAR binding to the PARP12-WWE domain. Thus, PAR formation functionally links the activity of the nuclear and cytosolic PARPs during stress response, determining the release of PARP12 from the Golgi complex and the disassembly of the Golgi membranes, followed by a block in anterograde-membrane traffic. Notably, these functions can be rescued by reverting the stress condition (by drug wash-out). Altogether these data point at a novel, reversible nuclear signaling that senses stress to then act on cytosolic PARP12, which in turn converts the stress response into a reversible block in intracellular-membrane traffic.

## Introduction

ADP-ribosylation (ADPR) is a post-translational modification of proteins involved in an expanding range of cellular functions and in the action of several toxins relevant for human pathology^1–3^; it consists of the enzymatic transfer of either a single ADP-ribose moiety (catalyzed by mono-ADP-ribosyltransferases), or multiple ADP-ribose units forming long and/or branched ADP-ribose polymers (catalyzed by poly-ADP-ribose-polymerases, PARPs) from NAD^+^ to specific residues on target proteins^4,5^. The modification of small molecules has also been reported, as in the case of the toxin brefeldin A, that can be enzymatically ligated to ADP-ribose, to then bind and inhibit a specific protein substrate (CtBP1-S/BARS; ref.^6^). In the last decade, new molecular tools for the study of the ADPR have become available^7–11^, and the mammalian ADP-ribosyltransferases have been systematically identified both at the genome and protein level^12^.

The human genome contains 22 genes that encode proteins with ADP-ribosyltransferase (ART) activity. ARTs are either ectoenzymes (ecto-ARTs) or intracellular enzymes. According to the structural features of their catalytic domains, these two families have been further sub-grouped into the cholera-toxin-like (ARTC) and the diphtheria-toxin-like (ARTD) ARTs, the latter also commonly known as PARPs^13^. The challenge is thus now to explore the regulation and the substrates and, in general, the role in cellular physiology and pathology of each ART^14–17^.

Here we focus on PARP12, a mono-ART of the PARP family, so far mainly studied as an interferon-stimulated gene, with roles both in the regulation of cell survival/regrowth and in the anti-viral response^18–22^. While the molecular mechanism responsible for cell survival is still unclear, PARP12-mediated anti-viral role has been linked to translational inhibition at both viral and cellular protein levels^19^. A similar role in translational shut-off as well as in the regulation of post-transcriptional gene expression has been also reported after ectopic expression of PARP12^22^, a condition known to induce the formation of stress granules^21^.

Stress granules are cytoplasmic, non-membranous, phase-dense structures that rapidly assemble in cells exposed to various types of stress including oxidative stress, heat or osmotic shock, and viral infection^23^; they persist until the damage is repaired, then disassembling within a few hours^24,25^. The fully formed stress granules are organized around the perinuclear region, where they act as hubs for a subset of signaling molecules that modulate metabolism, growth and survival^23,26^. Poly-ADP-ribose (PAR) polymer, a product of the poly-ADP-ribose-polymerases (PARPs), has been shown to be required in the cytoplasm for stress granule assembly in response to various stimuli^21^. Six PARP family members (PARP5a, -12, -13.1, -13.2, -14, -15) and two poly-ADP-ribose glycohydrolase (PARG) isoforms are known to localize at stress granules and to modulate, respectively, the kinetics of stress granule assembly and disassembly through the regulation of the cytosolic levels of PAR^21^.

In the following we report that PARP12 is a Golgi complex-localized mono-ADP-ribosyltransferase (mART) that upon stress translocates to stress granules. Importantly, stress-induced nuclear formation and release of PAR determines the recognition and binding to the PARP12 WWE domain, which precedes its translocation. Functionally, upon stress induction, PARP12 translocation associates with its loss of functions, with the fragmentation of the Golgi complex and impaired membrane transport. Of note, inhibition of PARP1-mediated PAR formation prevents PARP12 translocation to stress granules and partially rescues protein transport through the secretory pathway, suggesting that the translocation of PARP12 from the Golgi apparatus contributes to the impairment of intracellular trafficking as part of the stress response.

## Results

### The mART PARP12 translocates from the Golgi complex to stress granules upon stress conditions

**Figure 1:**
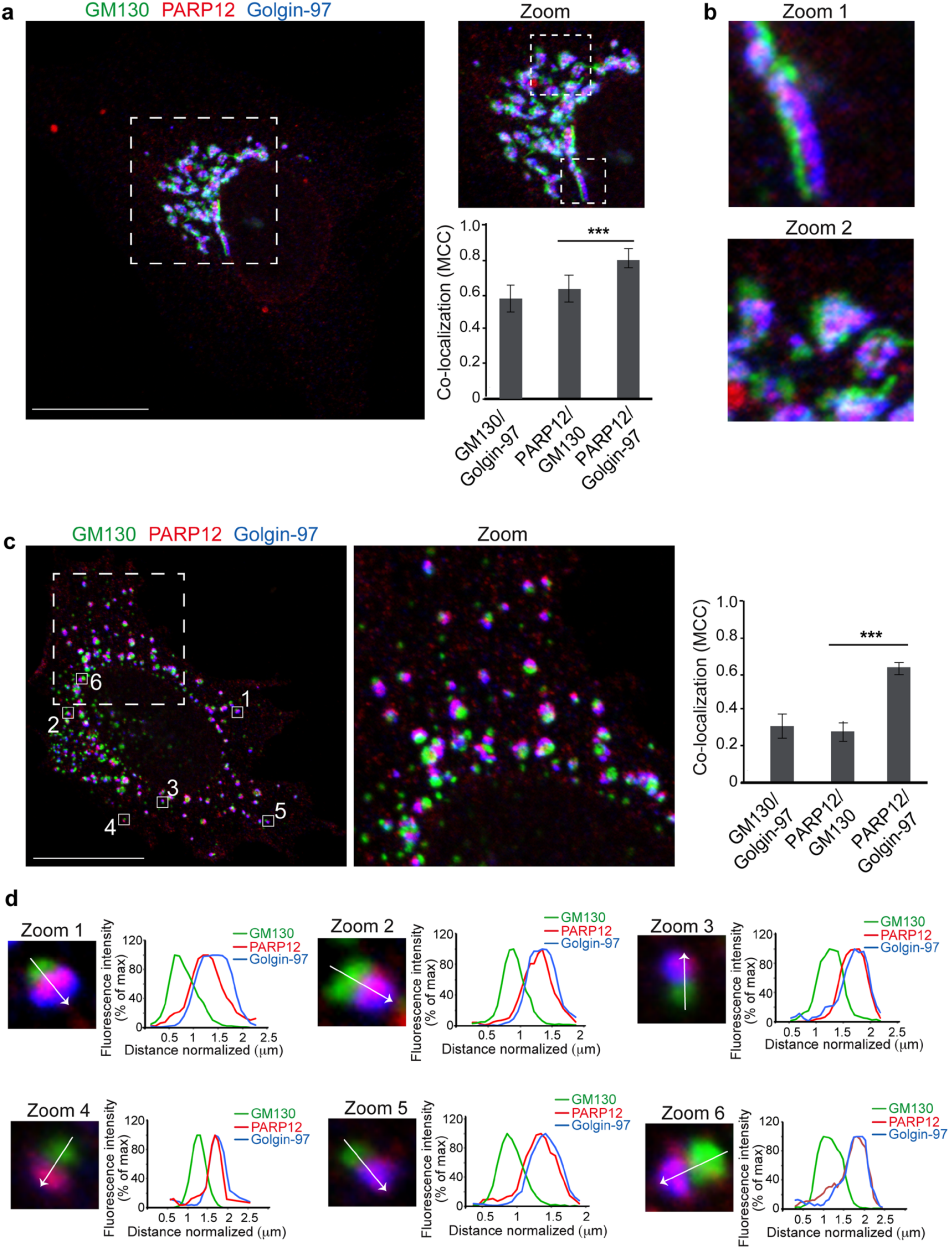
PARP12 is a *trans*-Golgi localized protein. (**a**) Representative confocal microscopy images of HeLa cells untreated (**a**,**b**) or treated with Nocodazole (33 μM, 3 h) (**c**,**d**), fixed and labeled with anti-PARP12 (red), anti-GM130 (a *cis*-Golgi marker, green) and anti-Golgin-97 (a TGN-Golgi marker, blue) antibodies. (**a**) Zoom: enlarged view of Golgi area. Quantification of Manders’ colocalization coefficient (MCC) of PARP12/GM130, PARP12/Golgin-97 and GM130/Golgin-97 (as internal control) are shown in the graph. Data are mean ± SD from *n* = 20. Statistical significance was calculated by unpaired Student’s *t*-test; ***p < 0.001. (**b**) Zoom 1 and 2: higher magnification images of Golgi area shown in **a**. (**c**) Clear separation of GM130 (green) and Golgin-97 (blue) markers in ministacks under Nocodazole treatment. Zoom: enlarged view of ministacks. Far right graph: quantification of Manders’ colocalization coefficient (MCC) under Nocodazole treatment. Data are mean ± SD from *n* = 20. Statistical significance was calculated by unpaired Student’s *t*-test; ***p < 0.001. (**d**) Zoom 1–6: enlarged view of single isolated mini stacks numbered in **c**. White arrow across the stack was used for line-scan analyses and the fluorescence intensity distribution of markers along this line was quantified and normalized to their respective peak values. The images shown are representative of three independent experiments. Scale bars, 10 μm.

To investigate the cellular functions of PARP12, we first examined its localization in HeLa cells using specific anti-PARP12 antibody and immunofluorescence microscopy. The endogenous PARP12 was visualized in the perinuclear area, where it co-localized with the *trans*-Golgi network (TGN) marker Golgin-97, but not with the *cis*-Golgi marker GM130 (Fig. 1a, b). This TGN localization was more evident after nocodazole-induced disruption of the Golgi ribbon (33 µM for 3 h) into smaller structures, the “mini-stacks”, known to be suitable to determine protein localization across the Golgi stacks^27,28^ (Fig. 1c; see also the fluorescence intensity profiles in Fig. 1d). PARP12 localized at the TGN, and not at the *cis*-Golgi, in all of the cell types tested (*e*.*g*., MDA breast cancer cells, THP leukemia monocytes and A375 melanoma cells; Supplementary Fig. 1). The TGN signal was abolished by PARP12 depletion, a proof of the antibody specificity (Supplementary Fig. 2).

**Figure 2:**
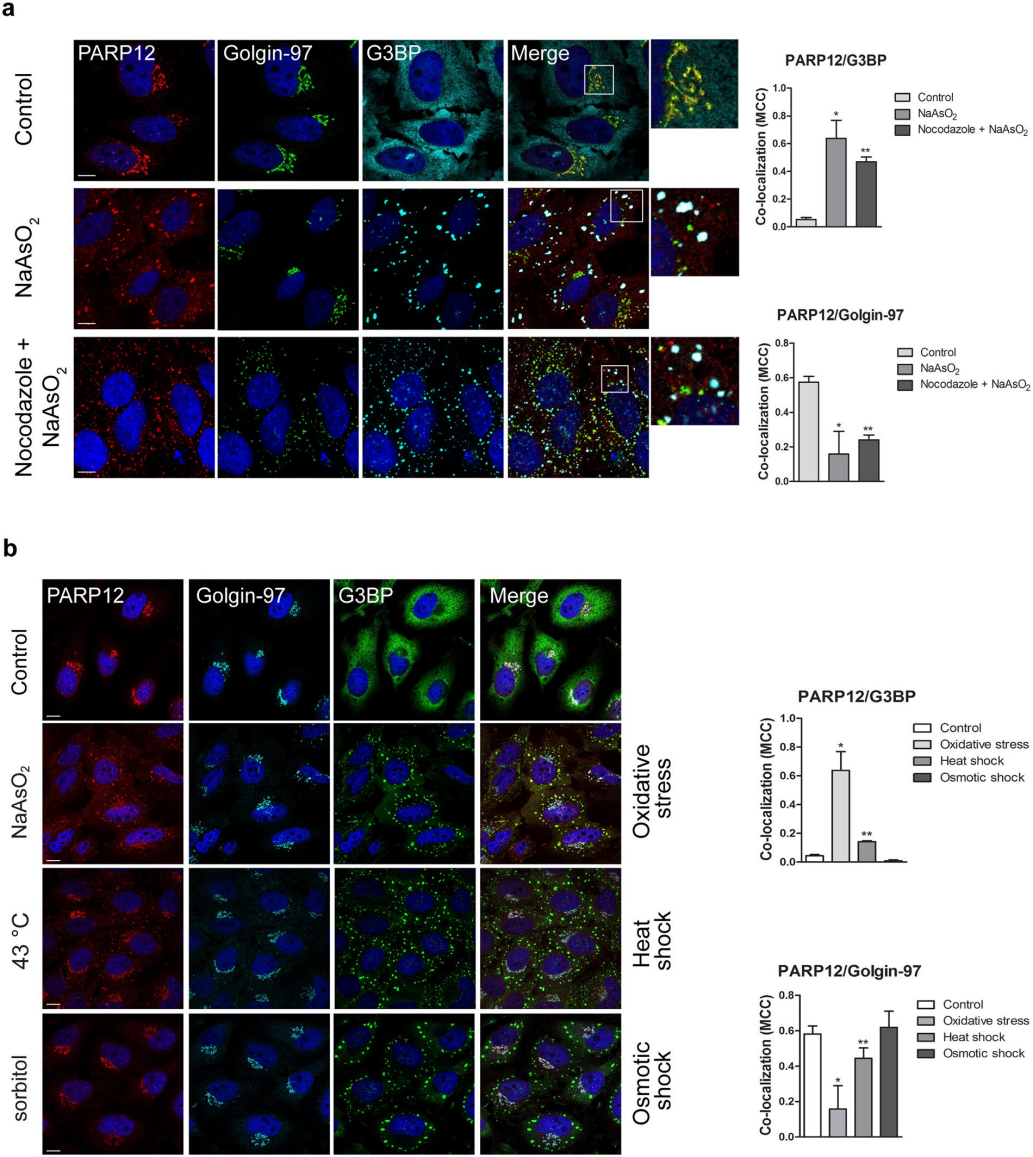
Redistribution of PARP12 from the Golgi complex to the stress granules under stress condition and in a microtubule independent manner. (**a**) Representative confocal microscopy images of HeLa cells untreated (Control) or treated with NaAsO_2_ alone (250 μM, 2 h; NaAsO_2_), or after Nocodazole treatment (33 μM, 3 h; Nocodazole + NaAsO_2_, Methods), fixed and labeled with anti-PARP12 (red), anti-Golgin-97 (green) and anti-G3BP (a stress granules marker; cyan) antibodies. Insets: enlarged view of merged signals. (**b**) Representative confocal microscopy images of HeLa cells untreated (Control) or exposed to oxidative stress (NaAsO_2_), heat shock (43 °C) or osmotic shock (sorbitol) (Methods), fixed and labeled as in **a**. (**a**,**b**) Far right graphs: quantification of Manders’ colocalization coefficient (MCC) of PARP12/G3BP and PARP12/Golgin-97 under the indicated treatments. Data are mean ± SD from three independent experiments. Statistical significance was calculated by unpaired Student’s t-test; *p < 0.05; **p < 0.01. No statistically significant difference was observed in the presence or in the absence of Nocodazole in NaAsO_2_ –treated cells. Scale bars, 10 μm.

PARP12 localization was further investigated under stress conditions known to induce stress granule formation^29^. Oxidative stress was induced by sodium arsenite addition (250 µM NaAsO_2_ for 2 h; Fig. 2a) that, as expected, promoted stress granule formation, and led to the translocation of endogenous PARP12 from the Golgi apparatus to stress granules, as indicated by its co-localization with the stress granule marker G3BP and decreased co-localization with the TGN marker Golgin-97 (Fig. 2a). A partial PARP12 translocation was detected at 30 min, when the protein was both Golgi-associated and cytosolic, and was complete after 2 h of stimulation (Supplementary Fig. 3).

Some stress granule components move along microtubules to reach these structures^30^. To evaluate whether this was also the case for PARP12, its traffic from the Golgi complex to stress granules was analyzed in cells pre-treated for 1 h with nocodazole (33 μM) before sodium arsenite addition. Under these conditions, a reduction in stress granule size and a change in their distribution were observed (as previously reported, ref.^24^), while no effect in PARP12 translocation towards stress granules was observed (Fig. 2a, as indicated by the quantification of the PARP12 and G3BP co-localization under nocodazole treatment), indicating that this process is independent of an intact microtubule network.

PARP12 localization at stress granules was also observed under stress induced by heat shock (cells were incubated at 43 °C for 45 min), but not when osmotic pressure was applied by addition of 1 M sorbitol to the growth medium (Methods and ref.^29^) (Fig. 2b), indicating that PARP12 is not an essential element for granule assembly. Since PARP12 translocation was better appreciable under oxidative stress induced by sodium arsenite (although the efficiency in stress granule formation with different stresses was similar, Supplementary Fig. 4) all the experiments reported herein refer to this condition.

Stress granules are dynamic structures that form within minutes of exposure to stress and dissolve within a few hours of stress recovery. Since PARP12 translocation is clearly dependent on the stress stimulus, we analyzed its localization also during stress recovery. HeLa cells were first treated with sodium arsenite and then allowed to recover from the stress in complete media for the indicated times. Under these conditions, PARP12 re-located at the Golgi complex as the stress was relieved, thus demonstrating the reversibility of its translocation (Supplementary Fig. 5).

Of note, we observed that oxidative stress caused a fragmentation of the Golgi apparatus. This phenotype was reversible (as indicated by a rescued Golgi phenotype after drug washout) and parallels the behaviour of PARP12 detachment from the Golgi complex (Supplementary Fig. 5).

Collectively, these data show that PARP12 is a TGN-localized protein, able to translocate to stress granules following oxidative stress, an event reversible upon stress relief; this change in localization parallels changes in the Golgi complex structure, suggesting that this latter event accompanies the stress response.

### Wild-type and mutant PARP12 trigger stress granule assembly independently of eIF2α modification

A feature shared by some stress granule components is their ability to nucleate stress granules after their overexpression, even in the absence of a stress stimulus^31^. This is the case also for PARP12 overexpression^21^, thus we investigated the contribution of PARP12 wild-type and its catalytically-inactive mutants raised in this study in the formation of stress granules.

The enzymatic activity of His-PARP12 isolated from Sf9 insect cells was characterized by following its auto-ADP-ribosylation (through the incorporation of [^32^P]-labeled ADP-ribose; Methods); the results were consistent with the identification of PARP12 as a mART of the PARP family (Supplementary Fig. 6 and its legend; see also refs^12,22^).

**Figure 3:**
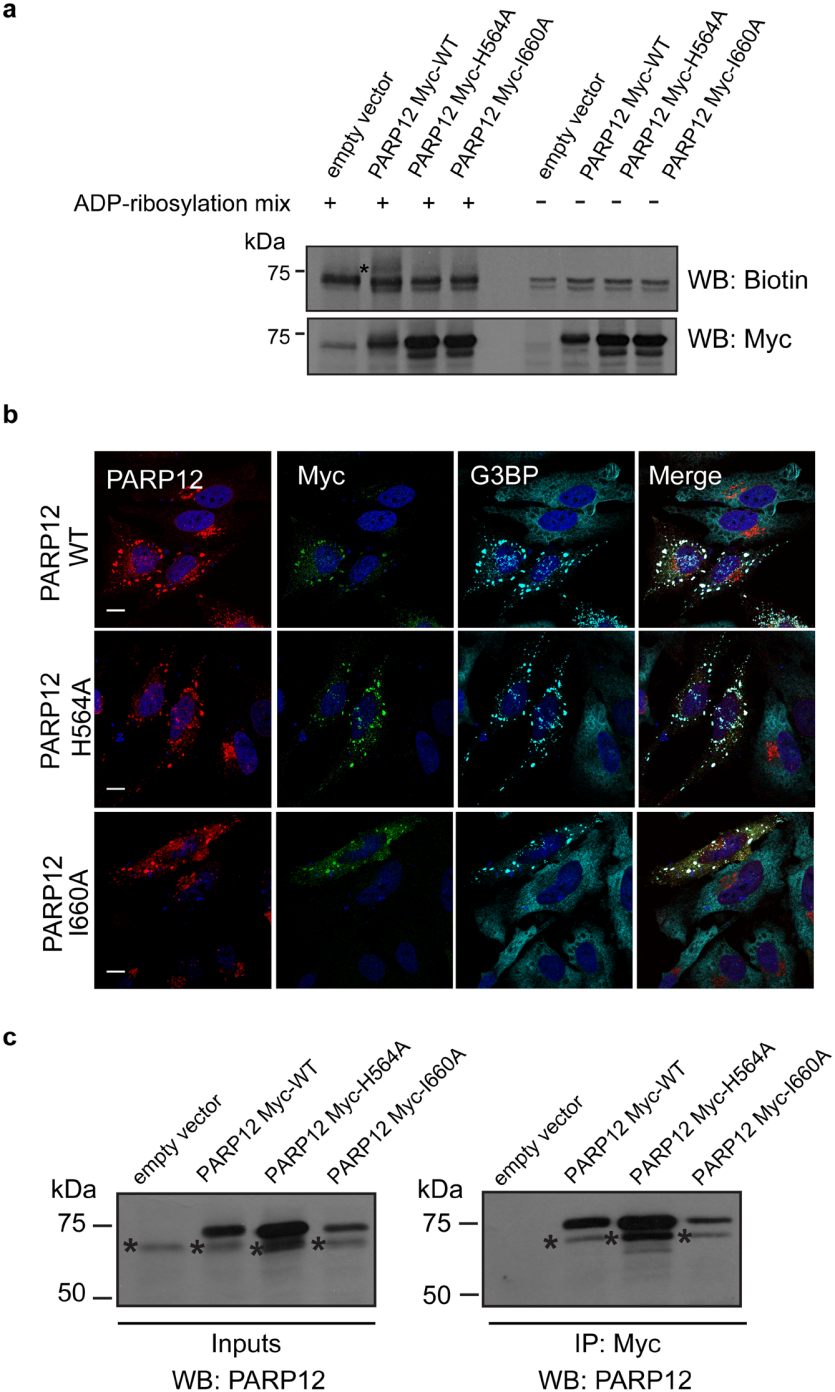
Overexpressed PARP12 induces stress granule assembly and interacts with its endogenous counterpart. (**a**) Representative *in vitro* ADP-ribosylation assay of total membranes from HeLa cells transfected with empty vector or Myc-tagged PARP12 wild-type (PARP12 Myc-WT) or different Myc-tagged PARP12 mutants (PARP12 Myc-H564A, PARP12 Myc-I660A) (Methods and Supplementary Table 1). The incorporated biotin-labeled ADP-ribose (black asterisk) was detected by Western blotting with anti-biotin antibody and the expression levels of PARP12 wild-type and mutants were monitored in membrane fractions with anti-Myc antibody. (**b**) Representative confocal microscopy images of HeLa cells transfected with empty vector or Myc-tagged PARP12 WT (PARP12 WT) or with the catalytically inactive mutants (PARP12 H564A, PARP12 I660A, as indicated) fixed and labeled with anti-PARP12 (red), anti-Myc (green) and anti-G3BP (cyan) antibodies. Scale bars, 10 μm. (**c**) Representative immunoprecipitation with anti-Myc antibody (IP: Myc) of total cell lysates from HeLa cells transfected with empty vector or PARP12 Myc-WT, PARP12 Myc-H564A or PARP12 Myc-I660A. Western blotting (WB) with anti-PARP12 antibody of eluted proteins (IP: Myc) and of total cell lysate (Inputs, 30 μg/lane). Black asterisks indicate endogenous PARP12. Molecular weight standards (kDa) in (**a** and **c**) are indicated on the left of each panel. Uncropped images of blots are shown in Supplementary Fig. 19.

PARP12 catalytic domain encloses the His-Tyr-Ile (H-Y-I) triad predicted to be required for its enzymatic activity^32^. Catalytically-inactive mutants were generated by substitution of the histidine 564 with either alanine or glutamine, and of the isoleucine 660 with alanine. The total membrane fractions of HeLa cells overexpressing either Myc-tagged PARP12 wild-type or the individual mutants were used as enzyme source to test the ADP-ribosylation activity in *in vitro* assays; as shown, the wild-type PARP12 incorporated biotin-labeled ADP-ribose, while the mutants did not, confirming the requirement of the H-Y-I for catalysis (Fig. 3a and Supplementary Fig. 7a).

We then examined the localization of these mutants following stress in HeLa cells transiently transfected with either Myc-tagged wild-type, or H564A/I660A catalytically-inactive mutants. PARP12 overexpression led to stress granule nucleation (Fig. 3b); under these conditions the endogenous protein translocated from the Golgi complex to these structures (Fig. 3b and Supplementary Fig. 7b). Endogenous PARP12 behaviour was similar to that of the overexpressed one. Indeed, this was further shown in experiments in which overexpressed Myc-tagged PARP12, wild-type and mutants, were immunoprecipitated using an anti-Myc antibody and revealed by western blot analysis using anti-PARP12 antibody. Figure 3c clearly indicates that the endogenous PARP12 (lower band) is in complex with both the wild-type and H564A/I660A catalytically-inactive mutants (having a higher molecular weight due to the presence of the tag). Thus, the recruitment of endogenous PARP12 to stress granules might result from the interaction/dimerization of the enzymes, followed by translocation.

Stress granules arise as a consequence of cellular stress and contain stalled translation pre-initiation complexes; this event can occur via both phosphorylation-dependent (phosphorylation of the α subunit of the eukaryotic initiation factor; eIFα) and -independent pathways that target translation initiation^33,34^. To explore the mechanism through which overexpression of PARP12 induced stress granules, we transfected HeLa cells with Myc-tagged PARP12 (wild-type and catalytically inactive mutants) and we evaluated by western blotting the phosphorylation of eIF2α at serine 51 (Ser51; Supplementary Fig. 8), a specific residue phosphorylated following diverse stress stimuli^25^. While, as expected, this phosphorylation was strongly induced by sodium arsenite treatment, no signal was detected after PARP12 overexpression (Supplementary Fig. 8), demonstrating that the assembly of stress granules through PARP12 overexpression is independent on eIF2α modification and thus may be activated by different pathways that target translation.

### The zinc finger and WWE domains of PARP12 determine its recruitment to stress granules: role of PAR

PARP12 shows a modular organization, with five N-terminal zinc finger domains (CCCH-type), two WWE domains, the PARP catalytic domain, and belongs to the sub-class of CCCH-PARPs together with PARP7 and PARP13^13^. We generated several PARP12 deletion mutants to define the role of its domain(s) both in PARP12 dimerization and in the recruitment to stress granules.

**Figure 4:**
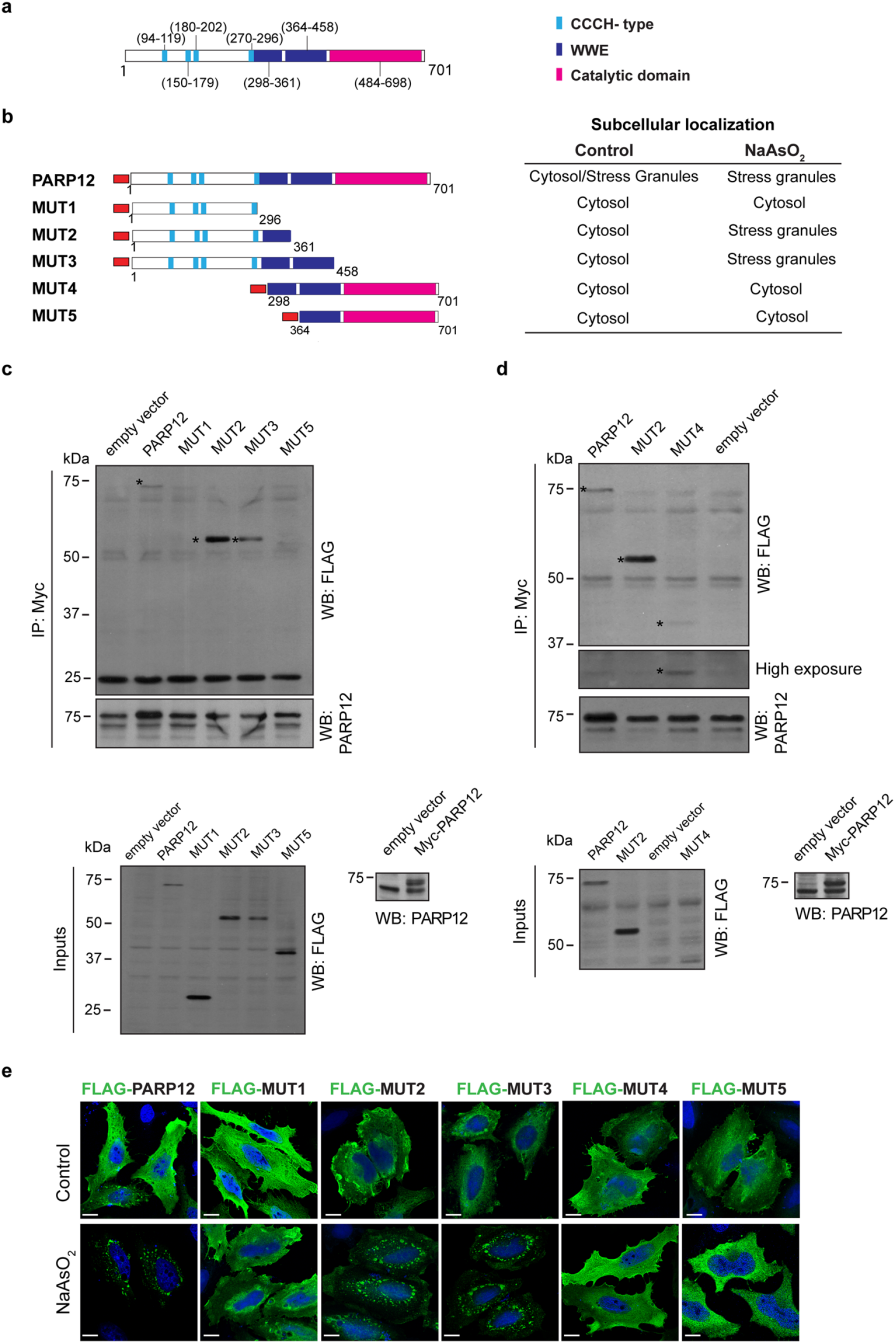
Recruitment of PARP12 deletion mutants to stress granules. (**a**) Structural domain organization of human PARP12, with functional annotation. The ZN-fingers (CCCH-type; light blue), the two WWE (blue) and the catalytic domains (magenta) are indicated. (**b**) Schematic representation of the PARP12 FLAG-tagged full-length (PARP12) and mutants (MUT1–5) used in this study and table summary of their subcellular localization in HeLa cells untreated (Control) or exposed to oxidative stress (NaAsO_2_). The FLAG-tag is indicated in red. (**c**,**d**) Immunoprecipitation with anti-Myc antibody (IP: Myc) of total cell lysates transfected with Myc-tagged PARP12 full-length followed by incubation with total cell lysates overexpressing FLAG empty vector, FLAG-tagged PARP12 full-length (PARP12) or the different PARP12-deletion mutants (as indicated). See Methods for details of lysis buffers used. The bound proteins were eluted and detected by Western blotting (WB) with anti-FLAG and anti-PARP12 antibodies (upper panels). Asterisks indicate the FLAG-tagged PARP12 proteins co-immunoprecipitated with Myc-tagged PARP12 protein. Total cell lysates (Inputs, 30 μg/lane) were also checked for overexpression levels with anti-FLAG antibody (lower panels; left) and with anti-Myc antibody (lower panels; right). Molecular weight standards (kDa) are indicated on the left of each panel. Uncropped images of blots are shown in Supplementary Fig. 19. (**e**) Representative confocal microscopy images of HeLa cells transfected with the FLAG-tagged PARP12 full-length (PARP12) or PARP12 deletion mutants (MUT1-5) for 24 h and then untreated (Control) or treated with NaAsO_2_ (250 μM, 2 h), fixed and labeled with anti-FLAG antibody (green). Scale bars, 10 μm.

Based on InterPro analysis that identifies the different domains in PARP12 sequence (accession Q9H0J9-PARP12 Human) (Fig. 4a), we generated five FLAG-tagged PARP12 deletion mutants corresponding to different portions of the protein: FLAG-PARP12 MUT1 (amino acids 1–296); FLAG-PARP12 MUT2 (amino acids 1–361); FLAG-PARP12 MUT3 (amino acids 1–458); FLAG-PARP12 MUT4 (amino acids 298–701); FLAG-PARP12 MUT5 (amino acids 364–701; Fig. 4b).

In order to analyze the specific region involved in the PARP12 dimerization, Myc-tagged full-length PARP12 was immunoprecipitated from total cell lysates and incubated with total cell lysates overexpressing FLAG-tagged wild-type full-length PARP12 or the different PARP12 deletion mutants, and the bound proteins were analyzed with an anti-FLAG antibody (Fig. 4c,d). As shown, only those mutants including the WWE1 domain were able to interact with the Myc-tagged full-length PARP12 (Fig. 4c,d), indicating that WWE1 domain is necessary for interaction.

In addition, the subcellular localization of the same PARP12 deletion mutants was analyzed by immunofluorescence in the absence or in the presence of oxidative stress. All of these mutants, in the absence of the stress stimulus, showed a cytoplasmic localization, while the addition of sodium arsenite led to the re-localization of FLAG-PARP12 MUT2 and MUT3 (the ones including the zinc finger domains *plus* at least the first WWE domain) at stress granules (Fig. 4e and Supplementary Fig. 9). Of note, FLAG-PARP12 MUT4 and MUT5 (having the WWE domains) were unable to translocate to stress granules upon oxidative stress (Fig. 4e). Taken together, these data demonstrate that the WWE domains are necessary but not sufficient for PARP12 localization at the stress granules and that this localization requires also the presence of the zinc finger domains (Fig. 4e).

**Figure 5:**
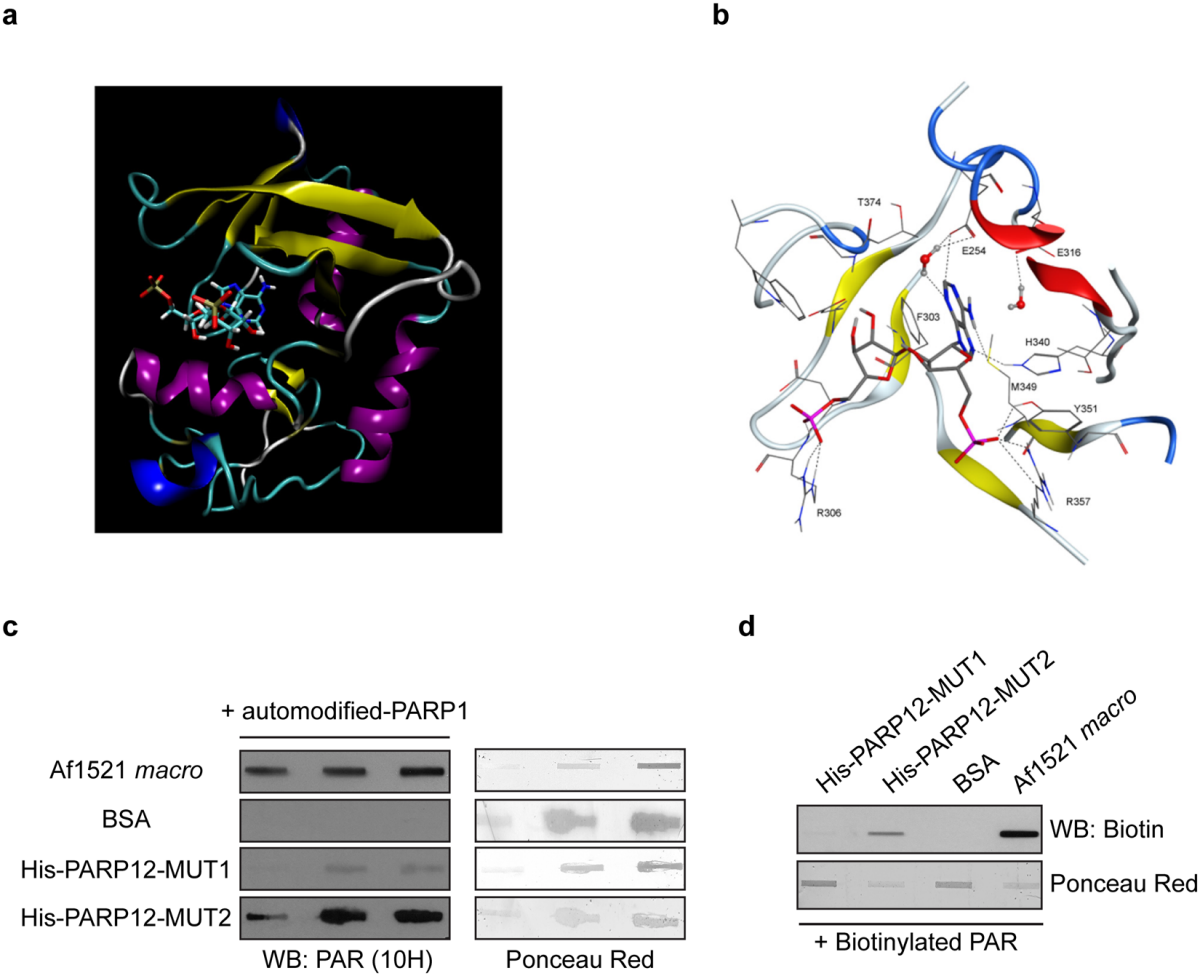
Homology model of the *iso*-ADP-ribose–PARP12 complex. (**a**) Homology model of PARP12 WWE1 using RNF146 crystal structure as template [(PMID: 25327252) PDBcode: 4QPL] with the *iso*-ADP-ribose molecules. The *iso*-ADP-ribose, in solid stick, is coloured per atom type, while the protein is schematized in a secondary-structure cartoon: alpha and 3.10 helices are in purple and blue, respectively; beta sheets are yellow arrows, beta turns are cyan ribbons and irregular structure are white ribbons. (**b**) Magnification of the ligand-binding site in PARP12 in presence of the *iso*-ADP-ribose as for the homology model in **a**. Engaged water molecules and key residue side chains are illustrated; main interactions stabilizing the complex are highlighted (dotted lines). Non-polar hydrogens are hidden. The *iso*-ADP-ribose and the interacting-residue side chains are coloured per atom type, while protein backbone is represented per secondary structure. Helices are in red, strands are in yellow, turns are in blue and ribbon loops are in white. (**c**,**d**) Far-Western blotting with purified His-PARP12-MUT1 and His-PARP12-MUT2 immobilized on a nitrocellulose membrane and incubated with PARP1-bound polymers as described under “Methods” (+automodified PARP1; **c**) or with biotinylated-PAR (+Biotinylated PAR; **d**) and revealed by Western Blotting with anti-PAR antibody [WB: PAR (10 H); **c**] or with anti-biotin antibody (WB: Biotin; **d**). Af1521 *macro* domain and BSA proteins were used as internal positive and negative controls, respectively. Ponceau Red staining was used to visualize the amount of proteins. Uncropped images of blots are shown in Supplementary Fig. 20.

Since PAR has been described to function as a scaffold molecule required for stress granule assembly^21^ and given the importance of the WWE domain(s) in supporting PARP12 translocation to these structures (Fig. 4e), we further investigated this aspect by a computational approach. To date, seven different WWE-domain crystal structures have been reported^35^ and, among them, the one from the E3 ubiquitin-protein ligase RNF146 (also known as Iduna) has been demonstrated to be involved in the binding of *iso*-ADP-ribose, the smallest structural unit of PAR^36^. RNF146 3D crystal structure (from *Mus musculus* [PMID:25327252] PDBcode: 4QPL) revealed that the *iso*-ADP-ribose binding site is composed by the zinc-finger RING type domain and the WWE domain. The inter-species WWE domain alignment (PROSITE database; PS50918) showed that both PARP12 WWE domains display a low sequence similarity with the RNF146 WWE region (Supplementary Fig. 10). Considering the specificities of WWE1 and WWE2 domains (see legend to Supplementary Fig. 10), we decided to address the homology modelling campaign on PARP12 WWE1, and to generate models using the RNF146 crystal structure as template (Methods). The homology model supports the conclusion that the PARP12 region defined by amino acids 226 to 377, including the WWE1 domain, is the binding site of the *iso*-ADP-ribose (Fig. 5a). Of note, in spite of the low sequence similarity between PARP12 and RNF146 WWE domains, several residues important for the interaction with *iso*-ADP-ribose are conserved. In details, Arg306 and Arg357 in PARP12 match the Arg110 and Arg161 of RNF146 (numbering of the latter residues refers to that of the crystal structure) and lock the *iso*-ADP-ribose into the WWE1 domain, orienting the molecule in the binding pocket (Fig. 5b). In addition to this key feature, other relevant interactions were preserved: i) the aromatic interaction (π-π stacking) between the Phe303 side chain (Tyr107 in the template) and the adenosine mojety of the *iso*-ADP-ribose; ii) two Hydrogen bonds established by His340 and Tyr351 (as done by Tyr144 and Arg155, respectively, in the template) with the imidazolic side of the adenosine head and with the phosphate group projected toward Arg357, respectively; iii) a water-bridged hydrogen bond further anchoring the adenosine moiety in the distinctly hydrophilic sub-pocket framed by the alpha helix 253–261, facing the WWE domain. Based on these considerations, we conclude that the zinc finger alpha helix and the WWE1 (amino acids 226–377) domain can be proposed as the pocket involved in the *iso*-ADP-ribose binding.

In line with this conclusion, we tested the *in vitro* interaction between the His-PARP12 MUT2 (spanning from amino acids 1–361, which includes the region identified by the above computational approach) and PAR. The recombinant purified His-PARP12 MUT2 was subjected to far-western blotting using PARP1-bound PAR (see Methods), or free biotinylated-PAR; His-PARP12 MUT2 was able to bind PAR, whereas His-PARP12 MUT1 under the same experimental conditions did not (Fig. 5c,d). Parallel experiments indicated that also MUT3 (spanning the region from amino acids 1 to 458) binds specifically PAR (Supplementary Fig. 11).

Altogether, these data indicate that PAR binding is part of the molecular mechanism that directs PARP12 to the stress granules.

### PAR is required for PARP12 re-localization from the Golgi complex to stress granules

**Figure 6:**
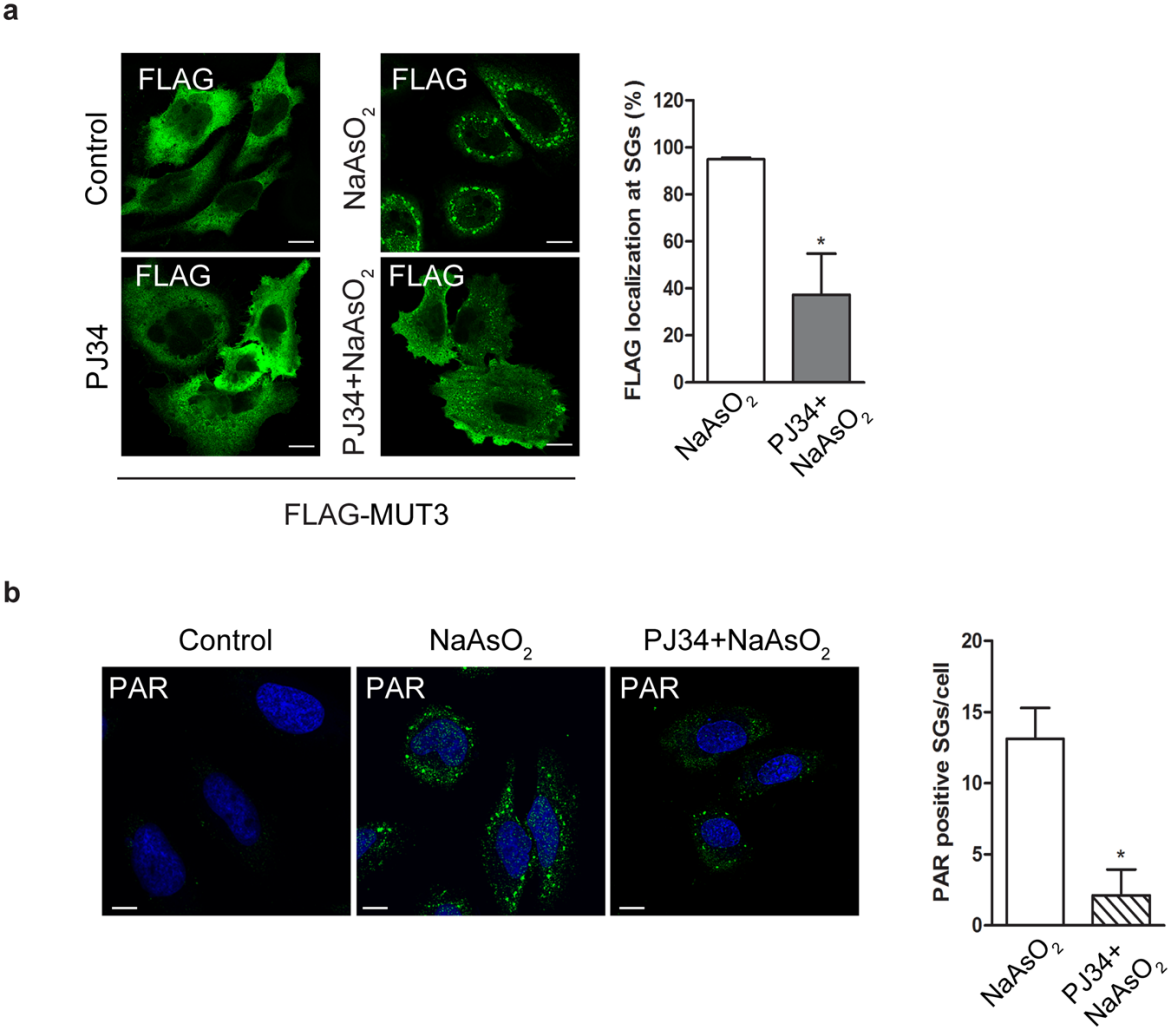
PAR triggers PARP12 translocation to stress granules. (**a**) Representative confocal microscopy images of HeLa cells transfected with FLAG-tagged PARP12-MUT3 (FLAG-MUT3) for 20 h and then untreated (Control) or treated with NaAsO_2_ (250 μM, 1 h) alone (NaAsO_2_), or with PJ34 pre-treatment (30 μM, 16 h; PJ34 + NaAsO_2_, Methods), before fixation and labeling with anti-FLAG antibody (green). Far right graph: quantification of cell percentage with FLAG-PARP12-MUT3 localization at stress granules (SGs). (**b**) Representative confocal microscopy image of HeLa cells untreated (Control) or treated with NaAsO_2_ alone (NaAsO_2_), or with PJ34 pre-treatment (PJ34 + NaAsO_2_; as above), fixed and labeled with anti-PAR antibody (green). Of note, the nuclear PAR staining is not detectable because the experiments were performed in the absence of PARG inhibitor (PDD00017238, 500 nM, 12 h). Far right graph: quantification of PAR positive stress granules/cell in NaAsO_2_- and (PJ34 + NaAsO_2_)-treated cells. Data are mean ± SD from three independent experiments. Statistical significance was calculated by unpaired Student’s t-test; *p < 0.05. Scale bars, 10 μm.

PAR is known to be localized at the stress granules where it is essential for their nucleation^21^. Our data emphasize a role of PAR also in the recruitment of PARP12 to the stress granules. This finding was further challenged by transiently transfecting HeLa cells with FLAG-PARP12 MUT3 (able to translocate to stress granules; see above) and analyzing its cellular localization as a function of PAR accumulation. After an overnight overexpression, HeLa cells were first treated with PJ34 (30 μM; the general PARP inhibitor able to reduce the enzymatic formation of PAR) and then exposed to oxidative stress (250 μM sodium arsenite, 1 h). Upon treatment with PJ34, before the stress challenge, the localization of the FLAG-PARP12 MUT3 at stress granules was clearly reduced (about 60%; Fig. 6a). In parallel, the PAR accumulation at these structures, evaluated by immunofluorescence analysis with a specific anti-PAR antibody, was also reduced (Fig. 6b). Under these conditions, a similar decrease was also observed for the translocation of endogenous PARP12 to stress granules (Supplementary Fig. 12).

Based on these data we conclude that PARP12 translocation from the Golgi complex to the stress granules is a PAR-dependent mechanism.

### PARP12 translocation from the Golgi complex to stress granules is dependent on nuclear PARP1 activation and PAR release

**Figure 7:**
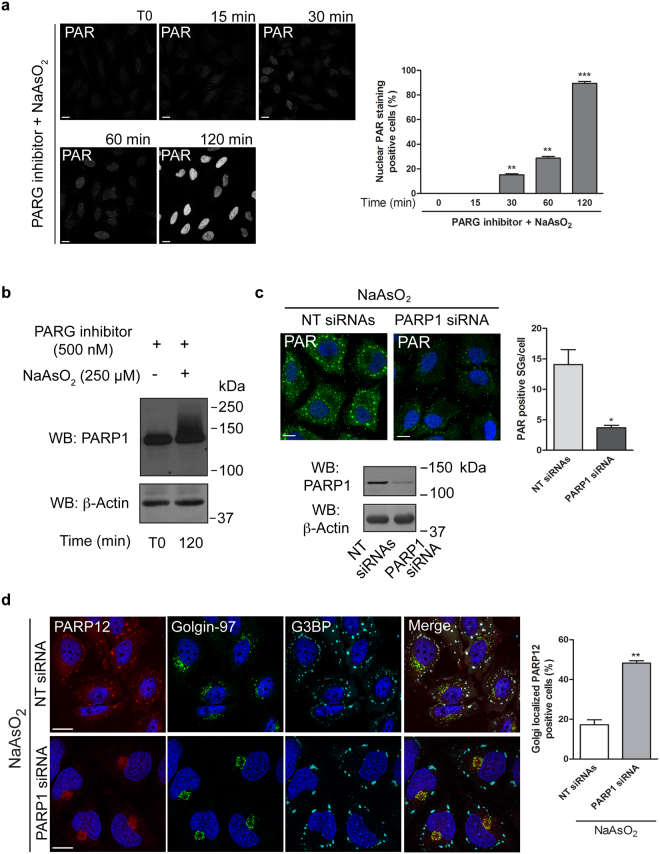
PARP1 activation drives PARP12 translocation to stress granules. (**a**) Representative confocal microscopy images of HeLa cells treated with PARG inhibitor (PDD00017238, 500 nM, 12 h) and then exposed to NaAsO_2_ (250 μM) for the indicated times, fixed and labeled with anti-PAR antibody (gray). Far right graph: quantification of cell percentage with nuclear PAR staining at the indicated times. Of note, in the absence of the oxidative stress, the nuclear PAR staining was not detectable at the analyzed times (from T0 to T120 min), even in the presence of the PARG inhibitor. (**b**) Western blotting (WB) with anti-PARP1 antibody of total cell lysate (20 μg/lane) from HeLa cells treated as in **a**. Uncropped images of blots are shown in Supplementary Fig. 20. (**c**) Representative confocal microscopy images of HeLa cells transfected with 150 nM non-targeting siRNAs (NT siRNAs) or with PARP1 siRNA for 72 h and then exposed to NaAsO_2_ (250 μM, 2 h), fixed and labeled with anti-PAR antibody (green). Far-right graph: quantification of PAR positive stress granules (SGs)/cell. Panel: efficiency of interference by Western blotting (WB) of total cell lysates (25 μg/lane) with anti-PARP1 antibody. (**b**,**c**) Actin is shown for the internal protein levels. Molecular weight standards (kDa) are indicated on the right of each panel. (**d**) Representative confocal microscopy images of HeLa cells treated as in **c**, fixed and labeled with anti-PARP12 (red), anti-Golgin-97 (green) and anti-G3BP (cyan) antibodies. Far right graph: quantification of cell percentage with PARP12 localization at the Golgi complex. Data are mean ± SD from three independent experiments. Statistical significance was calculated by unpaired Student’s t-test; *p < 0.05; **p < 0.01; ***p < 0.001. Scale bars, 10 μm.

Following on the central role of PAR in PARP12 recruitment to the stress granules, we next analyzed the potential source of PAR needed to support this function. The nucleus has been considered the major source of PAR due to the well-known activity of PARP1 and PARP2^37–39^. PARP1 can be activated during arsenite-induced oxidative stress and oxygen-radical formation, which causes DNA damage^40^. Therefore, we evaluated PARP1 activation following sodium arsenite addition by analyzing both nuclear PAR formation and PARP1 auto-ADP-ribosylation in cells treated with the PARG inhibitor (PDD00017238, 500 nM, 12 h) and then exposed to stress challenge (Fig. 7a). The accumulation of PAR (evaluated as above and quantified as the percentage of cells with nuclear PAR staining) upon sodium arsenite treatment was time-dependent and was evident in virtually all cells within 120 min (Fig. 7a). Under these conditions, and as expected, PARP12 translocated to the stress granules (Supplementary Fig. 13). PARP1 auto-ADP-ribosylation, detected by its molecular shift, occurred within the same time frame (Fig. 7b).

In order to assess the role of PARP1 activity in PARP12 translocation to stress granules, we inspected the enrichment of PAR at these structures in PARP1-knockdown cells under oxidative stress. Reduced accumulation of PAR at the stress granules was observed in PARP1-depleted cells (Fig. 7c). Similar results were obtained in cells pre-treated with selective PARP1/PARP2 inhibitors (30 μM ABT-888 or 30 μM Olaparib; Supplementary Fig. 14). In addition, endogenous PARP12 translocation to stress granules was impaired in PARP1-depleted HeLa cells (Fig. 7d). These results support the conclusion that PARP1 is the stress sensor and that it plays a key role in the recruitment of PARP12 to stress granules, by producing the PAR needed for such translocation. In line with this, the inhibition of nuclear PARP1 activity reduced endogenous PARP12 at the stress granules (Supplementary Fig. 12).

Following on the above findings we hypothesized that under oxidative stress nuclear PAR is transported to the cytoplasm and accumulates at the stress granules. Thus, cells pre-treated with the nuclear export inhibitor leptomycin B (LMB, 10 ng/ml, 16 h) were incubated with sodium arsenite for 2 h and both PAR accumulation and PARP12 localization were analyzed by immunofluorescence. Under these conditions, the PAR accumulation at stress granules and, as a consequence PARP12 translocation, were reduced (Supplementary Figs. 12 and 14).

In conclusion, PARP12 translocation from the Golgi apparatus to the stress granules depends on nuclear permeability and correlates with the PAR export from the nucleus to the cytoplasm, pointing at an essential role of PARP1 in the PARP12 localization and docking at these structures.

### The anterograde membrane transport is inhibited during oxidative stress

The observation that the stress challenge is followed by the fragmentation of the Golgi complex (see above) prompted us to analyze whether an impaired intracellular membrane trafficking was related to the stress response and/or represented a specific phenotype following stress.

**Figure 8:**
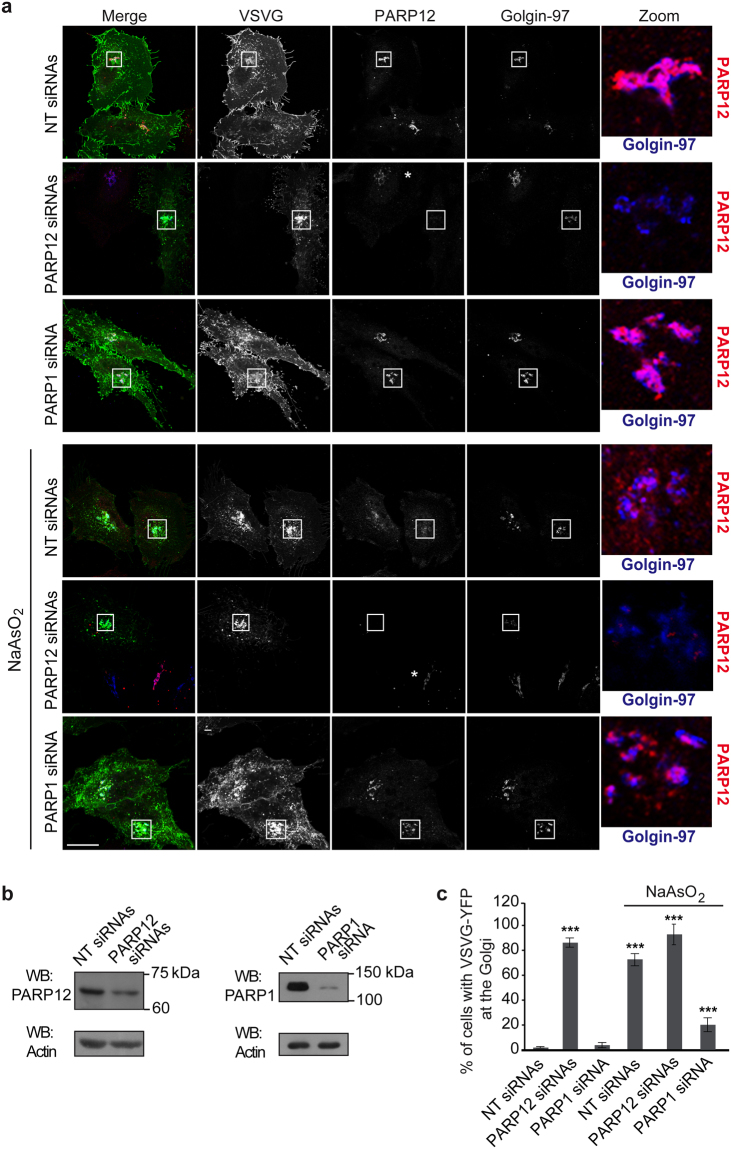
Oxidative stress impairs intracellular trafficking. (**a**) Representative confocal microscopy images of VSVG transport in HeLa cells transfected with 150 nM non-targeting siRNAs (NT siRNAs) or 100 nM PARP12 siRNAs or 150 nM PARP1 siRNA before co-transfection for the last 16 h with VSVG-YFP (Methods). During the TGN-exit assay, after 90 min at 20 °C, the cells were untreated or treated with 250 μM NaAsO_2_ (NaAsO_2_) and incubated for a further 90 min at 20 °C. Cells were fixed after 120 min of the 32 °C temperature-block release and labeled with anti-PARP12 (red) and anti-Golgin-97 (blue) antibodies. Insets: magnification of Golgi area. (**b**) Efficiency of interference by Western blotting (WB) of total cell lysates (25 μg/lane) with anti-PARP12 and anti-PARP1 antibodies, as indicated. Actin is shown for the internal protein levels and molecular weight standards (kDa) are indicated on the right of each panel. Uncropped images of blots are shown in Supplementary Fig. 20. (**c**) Quantification of VSVG-YFP in the Golgi area after 120 min of the 32 °C temperature-block release in cells treated as in **a**. Data are mean ± SD from three independent experiments. Statistical significance was calculated by unpaired Student’s t-test; ***p < 0.001 Scale bar, 10 μm. See also Supplementary Fig. 15 for cells fixed after the 20 °C temperature-block.

To evaluate these possibilities, we took advantage of a well-established transport assay of the temperature-sensitive variant of the vesicular stomatitis virus G protein (ts045-VSVG; for brevity, VSVG)^41^ to follow membrane trafficking under untreated or oxidative-stress conditions. Briefly, cells were transfected with YFP-tagged VSVG and incubated at 40 °C to first accumulate the protein in the endoplasmic reticulum. The cells were then shifted to 20 °C, a temperature that allows the exit of the cargo proteins from the endoplasmic reticulum and the arrival to, but not the exit from, the TGN. When cargo proteins were accumulated at the TGN, cells were treated or not with sodium arsenite. The temperature was finally shifted to 32 °C, and transport from the TGN to the plasma membrane was monitored by immunofluorescence (Fig. 8a and Supplementary Fig. 15). Sodium arsenite treatment inhibited transport from the TGN to the plasma membrane (anterograde traffic; Fig. 8a–c), indicating that the stress challenge impairs intracellular membrane traffic.

To examine the role of the Golgi-localized PARP12 in this process, we first analyzed the transport of VSVG in PARP12-depleted cells. Depletion of PARP12 had no effect on transport to the TGN (Supplementary Fig. 15), but it inhibited transport from the TGN to the plasma membrane (Fig. 8a–c and Supplementary Fig. 16). We next examined whether impairment in PAR formation, and thus in PARP12 translocation to stress granules with its retention onto the Golgi complex, could overcome the trafficking defect seen under stress conditions. To this purpose, the VSVG transport was analyzed in PARP1-depleted cells following sodium arsenite treatment (Methods). Depletion of PARP1 rescued the VSVG anterograde traffic, although not completely (by over 70%, compared to untreated cells; Fig. 8a–c). Accordingly, PARP12 was retained at the Golgi complex (Fig. 8a). Similar results were obtained using the PARP1 inhibitor Olaparib (100 μM, Methods and Supplementary Fig. 17).

Moreover, since the fragmentation of the Golgi complex following oxidative stress was shown to be reversible (Supplementary Fig. 5), we examined whether the Golgi stacks formed after drug wash-out were functional (Methods). Indeed, under these conditions VSVG was efficiently transported to the plasma membrane as in control cells, confirming the reversibility of the stress-induced traffic defect (Methods and Supplementary Fig. 18).

Altogether, these results show that the inhibition of PARP1-mediated nuclear signal (mimed in PARP1-depleted cells and in Olaparib-treated cells) partially rescues the membrane trafficking defect observed under sodium arsenite-induced stress, indicating that PARP12 contributes to the control of this reversible stress response.

In conclusion the stress challenge, in addition to the well established function in stress granule assembly and mRNAs translational arrest^25^, also affects Golgi complex structure with consequent impairment of the anterograde traffic.

## Discussion

In this study we show that the translocation of the Golgi-localized mART PARP12 to stress granules following oxidative stress is driven by the stress-induced activation of nuclear PARP1, resulting in the formation and release of PAR to the cytoplasm. Notably, cytosolic PAR is needed for PARP12 release from the Golgi membranes and for its localization at the stress granules, through its direct binding to PAR. We also show that under stress conditions the Golgi complex loses the classical ribbon morphology and, in parallel, anterograde membrane traffic is inhibited. Therefore PAR, that is known as a multifunctional compound involved in diverse cellular processes^42,43^, drives also the mechanism of PARP12 dissociation from the Golgi membrane and association to stress granules during stress.

These observations highlight a novel link between PARP1 function in the nucleus and PARP12 response at the Golgi membrane. Thus, the response to stress includes a reorganization of the Golgi complex (fragmented phenotype) that follows the PAR-mediated release of PARP12. PARP12 therefore behaves as a sensor of danger/stress, able to leave the Golgi membrane thus halting its function, while PAR is the signal leading this process. This is corroborated by the observation that the inhibitor of nuclear transport could prevent PARP12 translocation, as it did the PARP1 knock-down and inhibition (see Results).

This function of PARP12 depends on the poly-ADP-ribose-polymerase activity, in that it requires and follows the enzymatic formation of PAR by PARP1. No clear evidence of a contribution of the PARP12 enzymatic activity was observed in our study. Instead, a recent report shows that in Drosophila the response to amino acid starvation involves the dPARP16-mediated mono-ADP-ribosylation of the endoplasmic reticulum protein Sec16, necessary for Sec-bodies formation^44^; these are droplet-like structures, unrelated to stress granules, but representing a response to stress for cell survival^44^. Thus these lines of evidence indicate that both mono- and poly-ADP-ribose-polymerase activities might be involved in cell responses to different stresses.

Examples of connections between the nucleus and cellular organelles have been reported: DNA damage triggers a re-organization of the Golgi complex through the nuclear kinase DNA-PK-dependent phosphorylation of the Golgi-localized protein GOLPH3^45^; a pronounced DNA damage has been reported to induce PAR-dependent cell death (PARthanatos) through the nuclear import of Apoptosis Inducing Factor, that moves from the mitochondria to the nucleus following a PAR release in the cytoplasm^46,47^. Our data demonstrate a connection among nucleus, Golgi apparatus and stress granules, where PAR is a key signaling element during the stress response.

Free or protein-bound PAR polymers act as signal transducers by binding other proteins through their conserved PAR-binding modules^48^. PAR has been proposed as a novel organizer of cellular architecture: it aids the formation of non-membranous structures, such as DNA repair foci, spindle poles and RNA granules. Among these last structures, stress granules are particularly enriched in PAR, which serves as a hub for multiple proteins, including PARP12 and other PARPs, to bind in a non-covalent manner^49^.

Similarly to the other PARP family members, PARP12 has a multi-modular organization (Fig. 4a) and belongs to the CCCH-type subfamily together with PARP7 and PARP13. Recently, point mutations in the PARP12 zinc-finger domains were shown to prevent PARP12 translocation to the stress granules (a featured shared with PARP13, ref.^50^), implying the requirement of mRNA recognition by PARP12^22^. By analyzing PARP12 catalytically-inactive and deletion mutants we now show that PAR recognition by both its zinc-finger and WWE domains is strictly required for the recruitment to stress granules (see Results).

A novel observation emerging from this study is the impairment of anterograde membrane traffic during oxidative stress. It can be speculated that stress granules function as a platform to recruit not only components of the translational machinery, but also components of the secretory pathway to slow down its function during stress. PARP12 is part of this response, in that its translocation led to the inhibition of anterograde membrane traffic, an effect promptly rescued when PARP12 translocation was prevented by PARP1 depletion and inhibition (see Results). In addition, the microinjection of PAR in HeLa cells resulted in PARP12 release to the cytosol, suggesting that PAR displaces PARP12 from the membrane (preliminary results). Accordingly, we hypothesize that PARP1-mediated release of nuclear PAR controls PARP12 association/dissociation from the Golgi membrane also under physiological (non-stress) conditions.

A second example of Golgi complex-stress granules translocations upon oxidative stress is reported for the protein AKAP350A^51^, but the physiological consequences of this translocation remain unclear.

In conclusion, PARP12 can be considered an important mediator in cell response to stress: while it loses its localization at the Golgi complex contributing to the stall of intracellular trafficking, it acquires a different function at the stress granules, where it contributes to shutting-down translation^22^. This response to stress sees a central role of the PARP1/PAR-mediated nuclear signaling regulating the cytosolic PARP12 functions, thus adding to the interplay and multiple functions of the PARP family.

The molecular mechanisms underlying these PARP12 functions are still unclear; this becomes important in view of the involvement of these processes in degenerative disorders. Indeed, ribonucleoprotein (RNP) inclusions are part of the metabolism of eukaryotic mRNAs and are altered in several pathologies such as amyotrophic lateral sclerosis, fronto-temporal lobar degeneration^52,53^. An improved knowledge of the different stress granule components and of their roles during stress will be instrumental also for advancing our knowledge on these diseases and on potential therapeutic interventions.

## Methods

### Reagents and antibodies

Unless otherwise specified, all reagents were from Sigma-Aldrich. PARG inhibitor (PDD00017238) was kindly provided by I. Ahel (CRUK Drug Discovery Unit, Manchester); anti-Golgin-97 antibody by M.A. De Matteis (Tigem, Naples). [^32^P]-β-NAD^+^ (specific activity, 800 Ci mmol^−1^) was from PerkinElmer; Trichloroacetic acid from Carlo Erba; Paraformaldehyde from Electron Microscopy Sciences; Olaparib and ABT-888 from Selleckem; Biotinylated-PAR, biotinylated-NAD^+^ and recombinant purified PARP1 from Trevigen; Ni-NTA Agarose beads from Qiagen. Commercially available antibodies used: anti-PARP12 goat polyclonal (Abcam); anti-G3BP and anti-GM130 mouse monoclonal (BD Transduction Laboratory); anti-PAR monoclonal (Trevigen); anti-PAR (10 H) monoclonal (Enzo Life Sciences); anti-Myc (9E10 clone) monoclonal (Santa-Cruz); anti-Flag and anti-VSVG monoclonal (Sigma-Aldrich); anti-EIF2-α/phospho EIF2-α (Ser51) and anti-PARP1 polyclonal (Cell Signalling); anti-biotin (Bethyl Laboratories); HRP-conjugated streptavidin (Abcam), HRP-conjugated secondary antibodies (Calbiochem), HRP-conjugated anti-mouse secondary antibodies, Light Chain Specific (Jackson ImmunoResearch Laboratories); Alexa 488, 568 and 647 conjugated secondary antibodies (Molecular Probe). Recombinant PARP12 was purified from baculovirus infected Sf9 cells from Sigma-Aldrich. Human PARP12 cDNA was from Origene (for subcloning and mutations, see Supplementary Table 1).

### Cell culture, transfection and fractionation

HeLa, A375, MDA-MB-231 and THP cells were grown in MEM (supplemented with 100 μM MEM Non-Essential Amino Acids Solution), DMEM/F12, DMEM and RPMI-1640 respectively, with 10% (v/v) FBS (Biochrom), 2 mM L-glutamine, 50 U/ml penicillin and 50 μg/ml streptomycin. All cell culture reagents were from Life Technologies. HeLa cells were transfected: with plasmid encoding PARP12 wild-type or mutants or VSVG-YFP using TransIT-LT1 Reagent; with 100 nM PARP12 siRNA pool / L-013740-00-0005 (Dharmacon); with 100 nM PARP12 siRNA oligo 3 (5′-CGAAGAGCAUCCCAGACUA, Sigma-Aldrich); with 100 nM PARP12 siRNA oligo 4 (5′-CGAUGGCAAUUCUUGGAUA-3′, Sigma-Aldrich); with 150 nM PARP1 siRNA oligo 1 (5′-AAGCCTCCGCTCCTGAACAAT-3′, Sigma-Aldrich) or 150 nM PARP1 siRNA oligo 2 (5′-AACCCCAAAGGAATTCCGAGA-3′, Sigma-Aldrich) for 72 h using Lipofectamine 2000, according to manufacturer′s instructions. Total membrane fractions were prepared as described^6^.

### Induction of stress granules

Different stress stimuli were induced in HeLa cells by: addition of 250 μM NaAsO_2_ for 2 h (unless otherwise specified) in complete growth medium (oxidative stress); addition of 1 M sorbitol in complete growth medium for 30 min following cell wash and recovery for 1 h in complete growth medium without sorbitol (osmotic shock); incubation at 43 °C for 45 min in complete growth medium (heat shock). Where indicated cells were treated for 3 h with 33 μM nocodazole or for 16 h with 30 μM PJ34, or 30 μM Olaparib, or 45 μM ABT-888, or 10 ng/ml Leptomycin B (LMB).

### Immunofluorescence staining of PAR

PAR staining at stress granules was performed as described^54^, with modifications. HeLa cells were fixed first in methanol (5 min, −20 °C), washed twice in PBS, then fixed in 4% paraformaldehyde (10 min, RT), washed twice in PBS and permeabilized with 0.5% (v/v) Triton X-100 in PBS (5 min, RT). After three washes in PBS, cells were incubated with 100 mM sodium periodate (in 50 mM Tris-HCl pH 6.8) (5 min, RT), washed in PBS and further incubated with 25 mM succinic dihydrazide (in 50 mM Tris-HCl pH 6.8) (30 min, RT). Following washes in PBS, cells were incubated with 2% (v/v) normal goat serum blocking solution (2 h, RT) and with anti-PAR antibody (Trevigen) (16 h, 4 °C). After incubation with the primary antibody, cells were washed three times in PBS and incubated with a fluorescent-probe-conjugated secondary antibody (45 min, RT). Nuclear PAR staining was performed using the anti-PAR (10 H) antibody. Cells were pre-treated for 12 h with 500 nM PARG inhibitor (PDD00017238) and then treated with NaAsO_2_ (as described above). After 2 h incubation, cells were fixed in methanol/acetone (1:1 v/v) (10 min, −20 °C), washed three times in PBS, incubated in blocking solution [5% (w/v) non-fat dry skim milk in PBS supplemented with 0.05% (w/v) Tween 20] (1 h, RT) and then incubated with anti-PAR (10 H) antibody (2 h, RT). To follow PARP12 localization under these conditions, samples were incubated with anti-PAR (10 H) and anti-PARP12 antibodies (2 h, RT). After three washes in PBS, cells were incubated with fluorescent-probe-conjugated secondary antibodies (45 min, RT). Images were taken using a Zeiss-LSM 700 confocal microscope. Optical confocal sections were taken at 1 Air Unit. The co-localization analysis was performed using the JaCoP Plugin of the freeware ImageJ software according to the Manders’ Correlation Coefficient (MCC)^55^. For PAR-positive stress granule quantifications, images were analyzed using the freeware ImageJ software package, by automatically applying a threshold; the above-threshold fluorescent objects were then counted using the ‘Analyse Particle’ function. The co-localization analysis of Golgi mini stacks, with clearly separated GM130- and TGN-stained zones, was processed using Metamorph 7.7.3.0 (Universal Imaging). A line was drawn in the middle of the stacks along the cis-trans direction, and the fluorescence intensity of each stained marker along this line was plotted. The images obtained were then processed with the “Image with Zoom” function for presentation of Metamorph software.

### TGN-exit assay of VSVG

The TGN-exit assay of VSVG-YFP–transfected cells were carried out as reported^41^ with some modifications. HeLa cells were transfected with VSVG-YFP and incubated overnight at 40 °C following incubation at 20 °C for 90 min in presence of cycloheximide (50 μg/ml) to accumulate VSVG in the Golgi complex. Cells were then not exposed (control) or exposed to 250 μM NaAsO_2_ for a further 90 min at 20 °C to induce oxidative stress. To monitor VSVG exit from the TGN the temperature was shifted to 32 °C and the samples were fixed with 4% paraformaldehyde at the indicated times. Where indicated, at the end of the NaAsO_2_ treatment, cells were washed in complete growth medium and allowed to recover for the indicate times. For the assays performed in PARP1 depletion conditions, cells were transfected with 150 nM PARP1 specific siRNAs; after 48 h, cells were transfected with VSVG-YFP and processed as above. Alternatively, when the assay was performed in the presence of Olaparib, 100 μM final concentration of the inhibitor was added during the incubation at 20 °C for 3.5 h and for a further 1.5 h in the presence of 250 μM NaAsO_2_. At the end of 20 °C temperature block, cells were shifted in the presence of Olaparib at 32 °C and processed as above.

### ADP-ribosylation assay

PARP12 ADP-ribosyltransferase activity was determined by following the automodification of the protein, evaluated as incorporated [^32^P]-ADP-ribose. Purified His-PARP12 from Sf9 cells (500 ng) or total membrane fractions from HeLa cells overexpressing wild-type or catalytically inactive PARP12 mutants (50 μg) were incubated in 50 μl of ADP-ribosylation buffer [50 mM Tris-HCl pH 7.4, 4 mM DTT, 500 μM MgCl_2_, 30 μM unlabelled NAD^+^ /4 μCi of ^32^P-NAD^+^] at 30 °C for 30 min (purified His-PARP12) or at 37 °C for 2 h (membrane fractions). ADP-ribosylation inhibitors were pre-incubated with the enzyme (15 min, RT) and kept during the assay incubation-time at the indicated concentrations. ADP-ribosylation reactions with biotinylated NAD^+^ were performed with total membrane fractions from HeLa cells overexpressing wild-type or catalytically inactive PARP12 mutants (50 μg) and incubated in 50 μl of ADP-ribosylation buffer [50 mM Tris-HCl pH 7.4, 4 mM DTT, 500 μM MgCl_2_, 30 μM unlabeled NAD^+^ /20 μM biotinylated NAD^+^] at 37 °C for 2 h. The analysis of the ADP-ribosylated residue(s) was performed with the purified His-PARP12 as above and, at the end of the reaction time, the stability of the bound ADP-ribose assayed with 1 M NaOH or 100 mM HgCl_2_; alternatively, the reaction was performed in presence of the indicated concentration of meta-Iodobenzylguanidine (MIBG) for 2 h at 30 °C. The reactions were stopped by Laemmli buffer addition and samples were subjected to SDS-PAGE and transferred onto PVDF membranes. The incorporated [^32^P]-ADP-ribose was quantified using a Beta Imager instrument (Biospace Lab), while the incorporated biotin-labeled ADP-ribose was detected using anti-biotin antibody. Analysis of ADP-ribosylation reaction products was carried out as described^56^.

### PAR-Overlay Blot

The PAR binding assay was performed using automodified-PARP1 as PAR source. Briefly, purified PARP1 (200 ng) was incubated in 100 μl buffer containing 100 mM Tris-HCl pH 8.0, 10 mM MgCl_2_, 23 μg calf thymus activated DNA, 250 μM NAD^+^ at 25 °C for 2 h. The reaction was stopped by addition of 10 μM PJ34 and the mixture was then diluted 1:1000 in TTBS buffer [20 mM Tris-HCl, 0.05% (w/v) Tween 20, pH 7.5] for the binding assay. Purified His-PARP12 mutant proteins and controls were spotted using a Slot-Blot system (BioRad) onto a nitrocellulose membrane (cut-off 0.22 μm) and incubated with automodified-PARP1 (4 °C, 16 h). Non-specific binding was removed by high-stringency salt washes (3 times, 5 min, RT). Subsequently, blots were blocked in 5% (w/v) milk powder and protein-bound PAR was detected using anti-PAR (10 H) antibody. Alternatively, unfractionated biotinylated-PAR was used in PAR-binding assay as described^57^ with modifications. After incubation of the slot-blotted proteins with 1 μM biotinylated-PAR (4 °C, 16 h) and removal of non-specific binding, protein-bound PAR was detected using streptavidin-HRP (Abcam) or anti-biotin antibody. Af1521 *macro* domain, used as positive control, was purified as described previously^8^.

### Immunoprecipitation of PARP12 (wild-type and mutants)

For immunoprecipitation of Myc-tagged PARP12 (wild-type and mutants), HeLa cells in 10 cm plates were transiently transfected with plasmids using 18 μl TransIT-LT1 and 6 μg of DNA per plate. Twenty-four hours after transfection, cells were washed with PBS and lysed with RIPA Buffer [100 mM Tris-HCl pH 7.4, 150 mM NaCl, 0.1% (w/v) SDS, 1% (w/v) Igepal, 0.5% (w/v) sodium deoxycholate, supplemented with protease inhibitor cocktail]. Lysates were centrifuged (16,000 *g*, 20 min, 4 °C). Five hundred μg of protein lysate were incubated overnight with 2.5 μg mouse anti-Myc antibody. The mixtures were centrifuged (500 *g*, 5 min, 4 °C) and the beads were washed 3 times with RIPA buffer and further 3 times in the same buffer without detergents. The bound proteins were eluted by boiling (10 min) the beads in 100 μl Laemmli buffer, and then analyzed by SDS/PAGE and western blotting.

For interaction experiments between Myc-tagged PARP12 full-length and the FLAG tagged deletion mutants, HeLa cells in 10 cm plates were transiently transfected with FLAG-tagged constructs using 18 μl TransIT-LT1 and 6 μg of DNA per plate. Twenty-four hours after transfection, cells were washed with PBS and lysed with lysis buffer A [50 mM Tris-HCl pH 7.4, 150 mM NaCl, 1% (w/v) Triton-X100, supplemented with protease and phosphatase inhibitor cocktail]. For the FLAG-tagged MUT4 protein, cells were lysed using lysis buffer B [25 mM Tris-HCl pH 7.4, 150 mM NaCl, 1% (w/v) Triton-X100, 5 mM EDTA, 5 mM MgCl_2_ supplemented with protease and phosphatase inhibitor cocktail]. Lysates were centrifuged (16,000 *g*, 15 min, 4 °C). Seven hundred μg of protein lysate were incubated with immunoprecipitated Myc-tagged PARP12 wild-type (see above for the overexpression and immunoprecipation procedure) at 4 °C. After 2 h incubation, the mixtures were centrifuged (500 *g*, 5 min, 4 °C), the beads were washed 3 times with buffer A (or buffer B) and further 3 times in the same buffer without detergents. The bound proteins were eluted by boiling (10 min) the beads in 100 μl Laemmli buffer, and then analyzed by SDS/PAGE and western blotting.

### Expression and purification of His-tagged PARP12-Mutants

His-tagged PARP12-Mutants were generated from Human PARP12 cDNA from Origene as the pCMV6-XL5 using the oligonucleotides reported in Supplementary Table 1. The resulting pET28a plasmids encoding for His-PARP12-MUT1, His-PARP12-MUT2 and His-PARP12-MUT3 were used to transform *E*. *coli* strain BL21(DE3). The bacteria were grown in LB medium until they reached an OD_600_ nm of 0.6 and were induced by the addition of 0.5 mM IPTG for 16 h at 20 °C. The proteins were then purified on Ni-NTA matrix (Qiagen) according to the manufacturer’s instructions.

### Homology model

Homology models were generated using the Crystal structure of E3 ubiquitin-protein ligase RNF146 (RING-WWE) in complex with *iso*-ADP-ribose from *Mus musculus* [PMID:25327252] (PDBcode: 4QPL) as template. The amino acid sequences from the template and PARP12 (Q9H0J9 - PAR12_HUMAN) (region between 226 and 377 aa) were aligned using the bioinformatic tool MUltiple Sequence Comparison by Log-Expectation (MUSCLE) [PMID: 15034147]; then the alignment in the WWE region was manually optimized using the fingerprints of the WWE domain as aligned using in PROSITE or INTERPRO (PS50918 and IPR004170, respectively (http://prosite.expasy.org/PDOC50918). Due to the very low sequence identity between the modelled region and the template (sequence identity: 0.177) the proposed secondary structure was further analyzed by three different secondary structure prediction algorithms, namely Jpred, PsiPred and NetSurfP, which confirmed the reliability of the 3-D alignment. Homology modelling was performed with Modeller9.14 [PMID: 25199792], as implemented in BioVia Discovery Studio 4.1. The *iso*-ADP-ribose and water molecules within the binding site were taken into account. Twenty homology models were generated and the most promising one was selected according to DOPE and GA341 score and through a detailed visual inspection. Quality assessment of the selected model was achieved via stereochemistry measures such as the plot of angles depicted by Ramachandran plots using Molecular Operating Environment (MOE, 2013.08; Chemical Computing Group Inc.). The result obtained was parameterized with amber99 force-field as implemented in MOE, and 3-D protonation was applied to the system. Once visually inspected to avoid the presence of artefacts, the model underwent a multi-step minimization procedure: firstly, focusing on the binding site and then extended to the entire model. In details, a 6 Å radius sphere centred on the centre mass of the *iso*-ADP-ribose was considered to include all residues framing the ligand binding domain; this ensemble was minimized in two steps with and without backbone constraints. The same two steps approach was applied to the entire model to obtain the reported refined structure.

### Statistical analysis

Two-tailed Student’s t-tests were applied to the data. Significance is indicated as *P ≤ 0.05, **P ≤ 0.01 and ***P ≤ 0.001.

### Data availability

All data generated or analysed during this study are included in this published article (and its Supplementary Information files).

## Electronic supplementary material

Supplementary Information
