# Supplementary material for: PARP1-produced poly-ADP-ribose causes the PARP12 translocation to stress granules and impairment of Golgi complex functions
Source: Sci Rep. 2017 Oct 25;7:14035. doi: 10.1038/s41598-017-14156-8 (PMC5656619; doi:10.1038/s41598-017-14156-8)
Supplement: Supplementary file 1 — Supplementary Information [file 41598_2017_14156_MOESM1_ESM.pdf]

PARP1-produced poly-ADP-ribose causes the PARP12 translocation to stress granules and impairment of Golgi complex functions

Giuliana Catara[1], Giovanna Grimaldi[1,✉], Laura Schembri[1], Daniela Spano[1], Gabriele Turacchio[1], Matteo Lo Monte[1], Andrea Rosario Beccari[1,2], Carmen Valente[1,✉] and Daniela Corda[1,✉]

[1]Institute of Protein Biochemistry, National Research Council, Naples, Via Pietro Castellino 111, 80131, Italy

[2]Dompé Farmaceutici SpA Research Center, L'Aquila, Via Campo di Pile, 67100, Italy

Supplementary Table 1

TEMPLATE	OLIGONUCLEOTIDES	DIGESTIONS OF AMPLIFIED INSERTS	DESTINATION VECTOR
Human PARP12 cDNA from Origene as the pCMV6-XL5	5´- GTACGTAAGCTTATGGCCCAGGCCGGCGTCGT - 3´<br>5´- GTACGTCTCGAGCTGTCGGCTGCTGAACAGGG - 3´	*Hind*III/*Xho*I	*Hind*III/*Xho*I digested pcDNA3.1/myc-His vector (Invitrogen)
Human PARP12 cDNA from Origene as the pCMV6-XL5	**PARP12-FLAG-Full lenght**<br>5'-AAGCTTATGGCCCAGGCCGGCGTCGT-3'<br>5' -GGTACCTCACTGTCGGCTGCTGAACAGGGA-3'	*Hind*III/*Kpn*I	*Hind*III/*Kpn*I digested 3xFLAG CMV plasmid (Sigma)
Human PARP12 cDNA from Origene as the pCMV6-XL5	**PARP12-FLAG MUT1**<br>5'-AAGCTTATGGCCCAGGCCGGCGTCGT-3'<br>5'- GGTACCTCACCATGGTCAATGGAAATGAACTCT-3'	*Hind*III/*Kpn*I	*Hind*III/*Kpn*I digested 3xFLAG CMV plasmid (Sigma)
Human PARP12 cDNA from Origene as the pCMV6-XL5	**PARP12-FLAG MUT2**<br>5'- AAGCTTATGGCCCAGGCCGGCGTCGT-3'<br>5'- GGTACCTCAGGCCGTGGAGAGGCGGCGA-3'	*Hind*III/*Kpn*I	*Hind*III/*Kpn*I digested 3xFLAG vector (Sigma)
Human PARP12 cDNA from Origene as the pCMV6-XL5	**PARP12-FLAG MUT3**<br>5'- AAGCTTATGGCCCAGGCCGGCGTCGT-3'<br>5'- GGTACCTCAGCGGCAAACCTTTTTAGTTGT-3'	*Hind*III/*Kpn*I	*Hind*III/*Kpn*I digested 3xFLAG vector (Sigma)
Human PARP12 cDNA from Origene as the pCMV6-XL5	**PARP12-FLAG MUT4**<br>5'-AAGCTTATGTATCGATGGCAATTCTTGGATAGA-3'<br>5'-GGTACCTCACTGTCGGCTGCTGAACAGGGA-3'	*Hind*III/*Kpn*I	*Hind*III/*Kpn*I digested 3xFLAG vector (Sigma)
Human PARP12 cDNA from Origene as the pCMV6-XL5	**PARP12-FLAG MUT5**<br>5'-AAGCTTGGTGCTACCCAGGCTCG-3'<br>5' -GGTACCTCACTGTCGGCTGCTGAACAGGGA-3'	*Hind*III/*Kpn*I	*Hind*III/*Kpn*I digested 3xFLAG vector (Sigma)
Human PARP12 cDNA from Origene as the pCMV6-XL5	**PARP12 HIS-MUT1**<br>5'-CATGTCGTCGGTGAGGTCACCCAGGTGCT-3'<br>**5'-**GCGGCCGCTCACAAATGGAAATGAACTCT-3'	*Nde*I/*Not*I	*Nde*I/*Not*I digested pET28a vector (Novagen)
Human PARP12 cDNA from Origene as the pCMV6-XL5	**PARP12 HIS-MUT2**<br>5'-CATGTCGTCGGTGAGGTCACCCAGGTGCT-3'<br>**5'-**GCGGCCGCTCAGGCCGTGGAGAGGCGGCGA-3'	*Nde*I/*Not*I	*Nde*I/*Not*I digested pET28a vector (Novagen)
Human PARP12 cDNA from Origene as the pCMV6-XL5	**PARP12 HIS-MUT3**<br>5'-CATGTCGTCGGTGAGGTCACCCAGGTGCT-3'<br>5'-GCGGCCGCTCATCAGCTGGGGGCAGAAGCTCTTA TTTCTT-3'	*Nde*I/*Not*I	*Nde*I/*Not*I digested pET28a vector (Novagen)
Human PARP12 cDNA in pcDNA3.1/myc-His vector	**PARP12 H564A**<br>5'- CGAGCGGCAGCTGTTCGCCGGCACCAGCGCCATT TTT-3'<br>5'-AAAAATGGCGCTGGTGCCGGCGAACAGCTGCCGC TCG-3'		
	**PARP12 H564Q**<br>5'- CGAGCGGCAGCTGTTCCAAGGCACCAGCGCCATT TTT-3'<br>5'- AAAAATGGCGCTGGTGCCTTGGAACAGCTGCCGC TCG-3'		
	**PARP12 I660A**<br>5'- GAACAGTGTGTCCGACCCCTCCGCCTTTGTGATCT TTGAGAAA-3'<br>5'- TTTCTCAAAGATCACAAAGGCGGAGGGGTCGGAC ACACTGTTC-3'		

Supplementary Table 1. List of the oligonucleotide sequences for amplification of the templates, and of the restriction enzymes used in the cloning of the listed expression vectors.

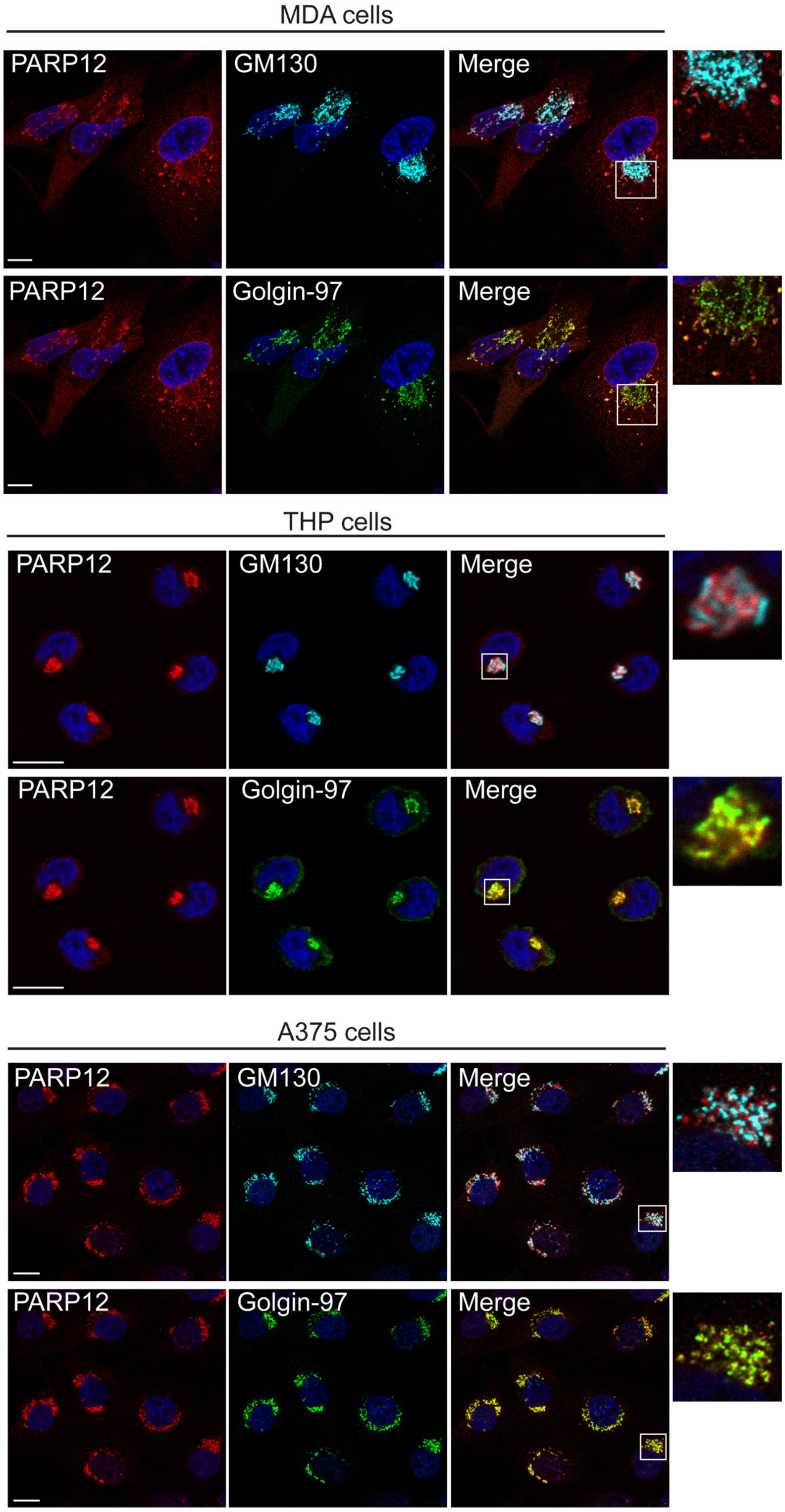

Supplementary Fig. 1. PARP12 localization at the Golgi complex in MDA, THP and A375 cells. Representative confocal microscopy images of MDA, THP and A375 cells (as indicated) fixed and labeled with anti-PARP12 antibody (red) and anti-GM130 (cyan) or anti-Golgin-97 (green) antibodies. Insets: enlarged view of the Golgi area. Scale bars, 10 μm.

Supplementary Fig. 2

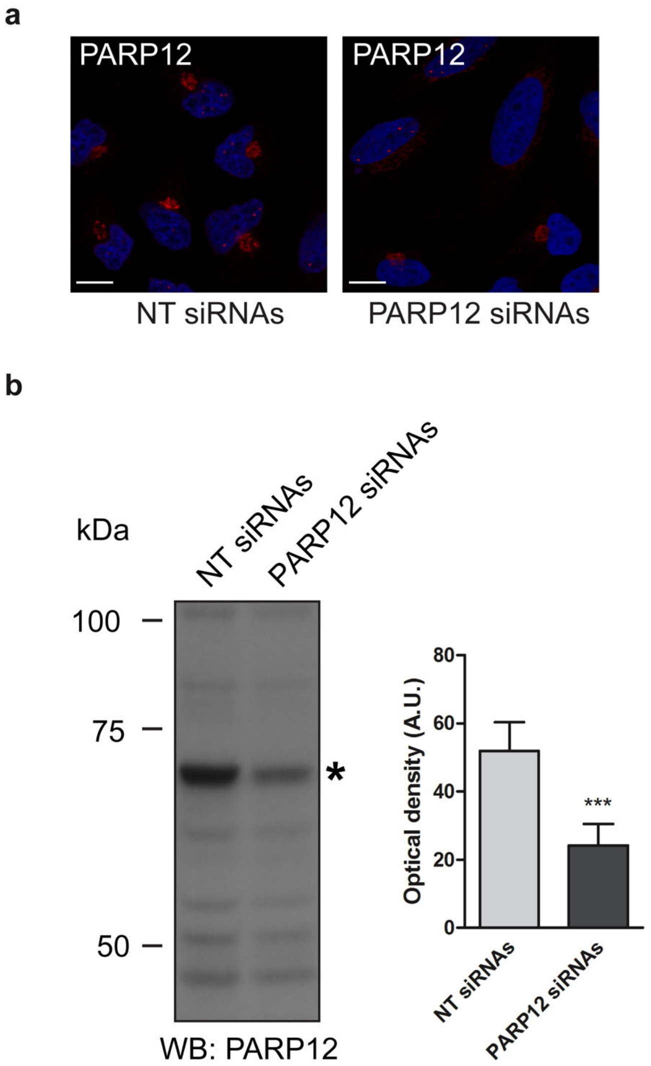

Supplementary Fig. 2. Specificity of the PARP12 antibody. (**a**) Representative confocal microscopy images of HeLa cells transfected with 100 nM non-targeting (NT siRNAs) or with PARP12 siRNAs for 72 h, fixed and labeled with anti-PARP12 antibody (red). Scale bars, 10 µm. (**b**) Efficiency of interference and specificity of the anti-PARP12 antibody by Western blotting (WB) of cell lysates (treated as indicated; 30 µg/lane). Asterisk indicates the PARP12 specific band. Molecular weight standards (kDa) are indicated on the left of the panel. Quantification of PARP12 depletion level (PARP12 siRNAs) compared to control (NT siRNAs) cells is shown in the graph as mean ±SD from five independent experiments. Statistical significance was calculated by unpaired Student's t-test; ***$p < 0.001$.

Supplementary Fig. 3

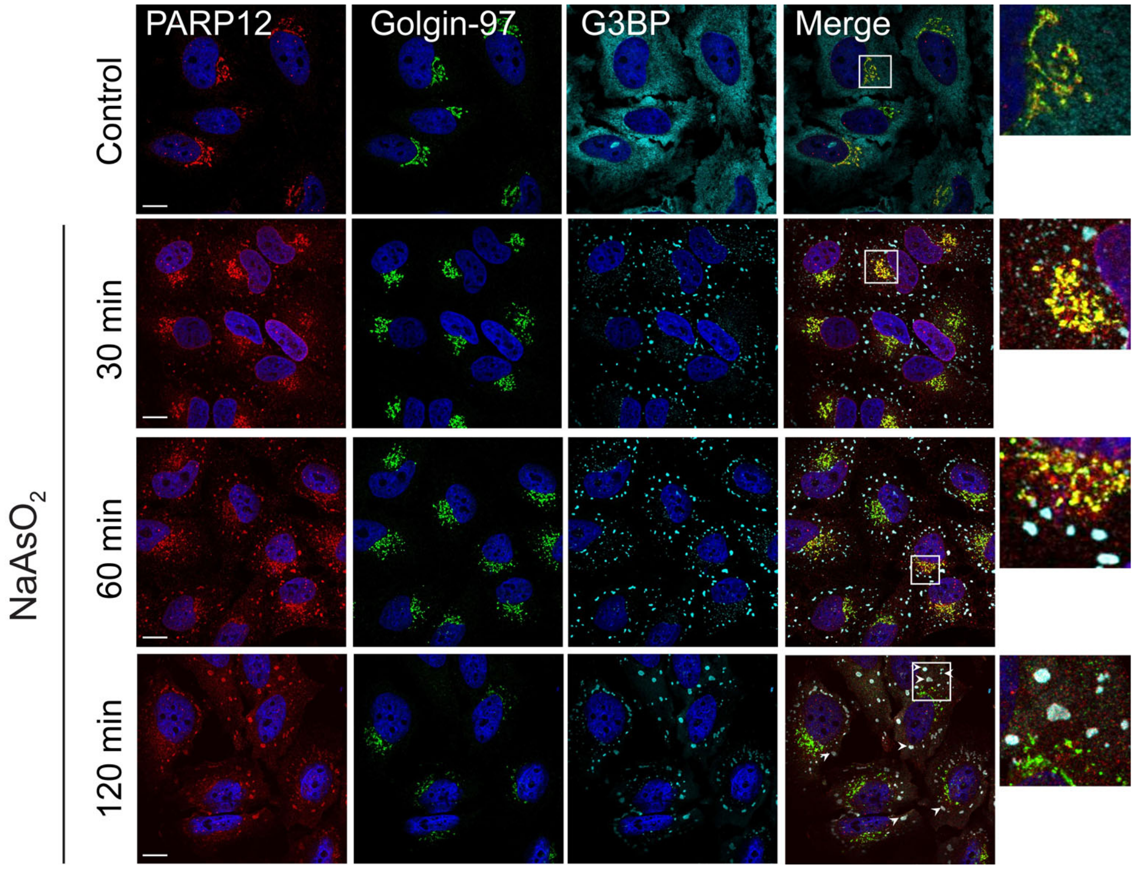

Supplementary Fig. 3. PARP12 redistribution to stress granules upon oxidative stress. Representative confocal microscopy images of HeLa cells untreated (Control) or treated with 250 μM of $NaAsO_2$ for 30, 60 and 120 minutes (as indicated) fixed and labeled with anti-PARP12 (red), anti-Golgin-97 (green) and anti-G3BP (cyan) antibodies. White arrowheads refer to protein localization at stress granules. Insets: enlarged view of the Golgi area (top panel) and stress granules (bottom panel). Scale bars, 10 μm.

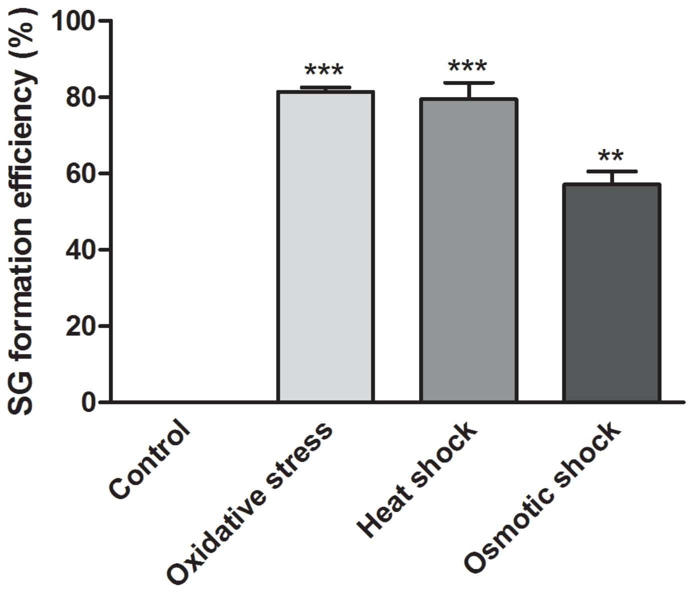

Supplementary Fig. 4. Efficiency of stress granule formation. HeLa cells untreated (Control) or exposed to oxidative stress (250 μM, 2 h; $NaAsO_2$), heat shock (43 °C) or osmotic shock (sorbitol) (Methods), were fixed and labeled with anti-G3BP (a stress granules marker) antibody. Efficiency in stress granule formation was quantified by analyzing the percentage of cells showing stress granules upon stress induction. Statistical significance was calculated by unpaired Student's t-test; $**p < 0.01$; $p *** < 0.001$.

Supplementary Fig. 5

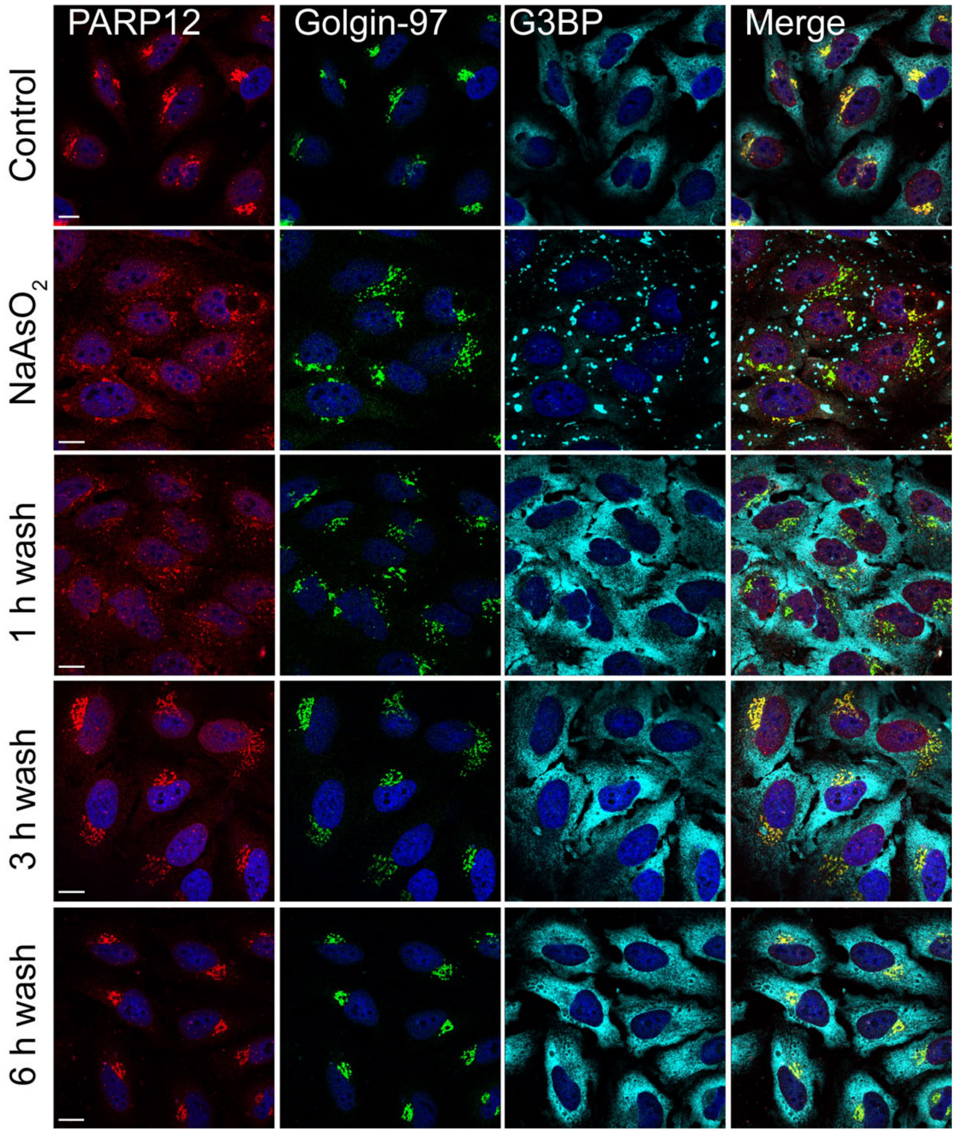

Supplementary Fig. 5. PARP12 translocation from the Golgi complex to stress granules is reversible. Representative confocal microscopy images of HeLa cells untreated (Control) or treated for 2 h with 250 µM of $NaAsO_2$ and fixed ($NaAsO_2$) or additionally incubated in complete media without $NaAsO_2$ at 37 °C for the indicated times (1 h, 3 h or 6 h wash) followed by fixation and labeling with anti-PARP12 (red), anti-Golgin-97 (green) and anti-G3BP (cyan) antibodies. Scale bars, 10 µm.

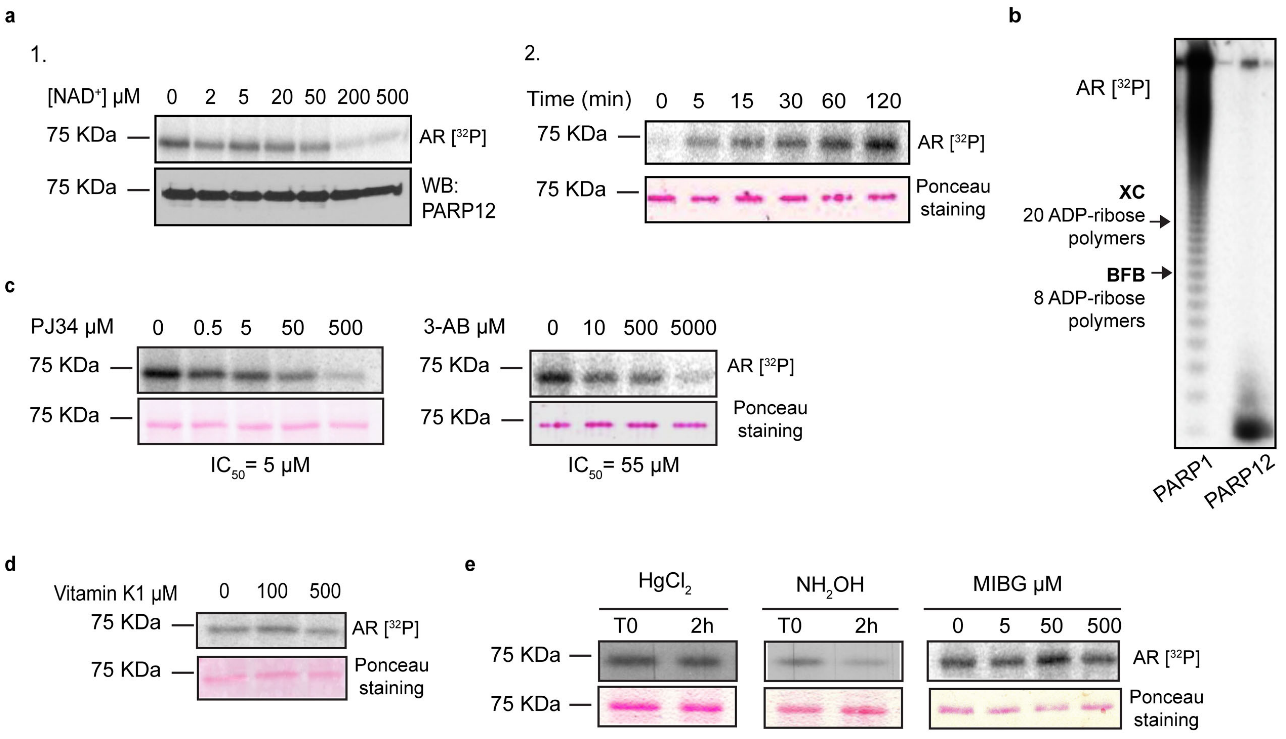

Supplementary Fig. 6. Characterization of PARP12 mono-ADP-ribosyltransferase activity. (**a**) ADP-ribosylation assay of His-PARP12 (purified from Sf9 insect cells, Methods) incubated with 4 µCi of [^{32}P]-NAD^{+} and increasing concentrations (as indicated) of unlabeled NAD^{+} for 30 min at 30 °C (panel 1) or with 30 µM of total NAD^{+} and 4 µCi of [^{32}P]-NAD^{+} and stopped at different times (panel 2). PARP12 protein levels were analyzed by Western blotting using anti-PARP12 antibody (WB: PARP12, panel 1) or by Pounceau staining (panel 2). The incorporated [^{32}P]-ADP-ribose was detected by autoradiography (AR[^{32}P]). Of note, the ADP-ribosylated PARP12 did not show any change in mobility as a function of substrate concentrations or incubation times. (**b**) Polymer chain length analysis (Methods) revealed by autoradiography (AR[^{32}P]) of *in vitro* ADP-ribosylated PARP1 and PARP12 proteins. The ADP-ribose moieties were removed from the proteins by base-treatment and analyzed on a sequencing gel. Arrowheads indicate the length of ADP-ribose chains determined by co-migration with bromophenol blue (BFB, 8-ADP-ribose polymers) and xylene cyanol (XC, 20-ADP-ribose polymers). Of note, while PARP1 catalyzed the formation of longer polymers, monomeric ADP-ribose moiety was detectable after PARP12 auto-modification in agreement with its classification as mART. (**c**, **d**) ADP-ribosylation assays, as described in **a**, of His-PARP12 performed in presence of different ADP-ribosylation inhibitors: PJ34 and 3-AB (**c**) or Vitamin K1 (**d**) at different concentrations (as indicated). The incorporated [^{32}P]-ADP-ribose was

detected by autoradiography (AR[^{32}P]) and PARP12 protein levels were analyzed by Ponceau staining. PARP12 was inhibited by PJ34 and by 3-AB, two wide-range PARP inhibitors, with IC_{50} values of 5 μM and 55 μM, respectively, whereas no effect of Vitamin K1, a classical inhibitor of ecto mARTs, was detected. (**e**) ADP-ribosylation assays of His-PARP12 incubated for 30 min with 30 μM of total NAD^+ and 4 μCi of [^{32}P]-NAD^+ before the addition of 100 mM of $HgCl_2$ or 1 M of NH_2OH for 0 or 2 h, or the addition of meta-Iodobenzylguanidine (MIBG) at the indicated concentrations for 2 h. The incorporated [^{32}P]-ADP-ribose was detected by autoradiography (AR[^{32}P]) and PARP12 protein levels were analyzed by Ponceau red staining. Molecular weight standards (kDa) are indicated on the left of each panel. Uncropped images of blots are shown in Supplementary Fig. 21.

Supplementary Fig. 7

a

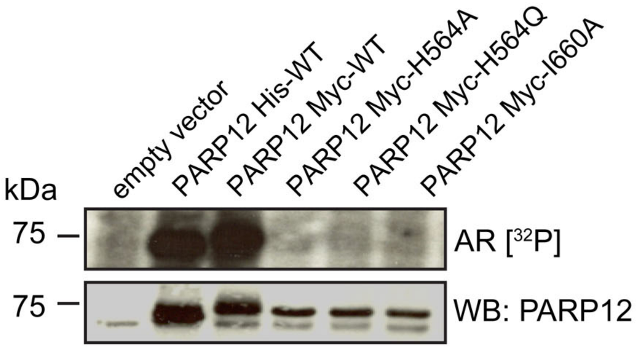

b

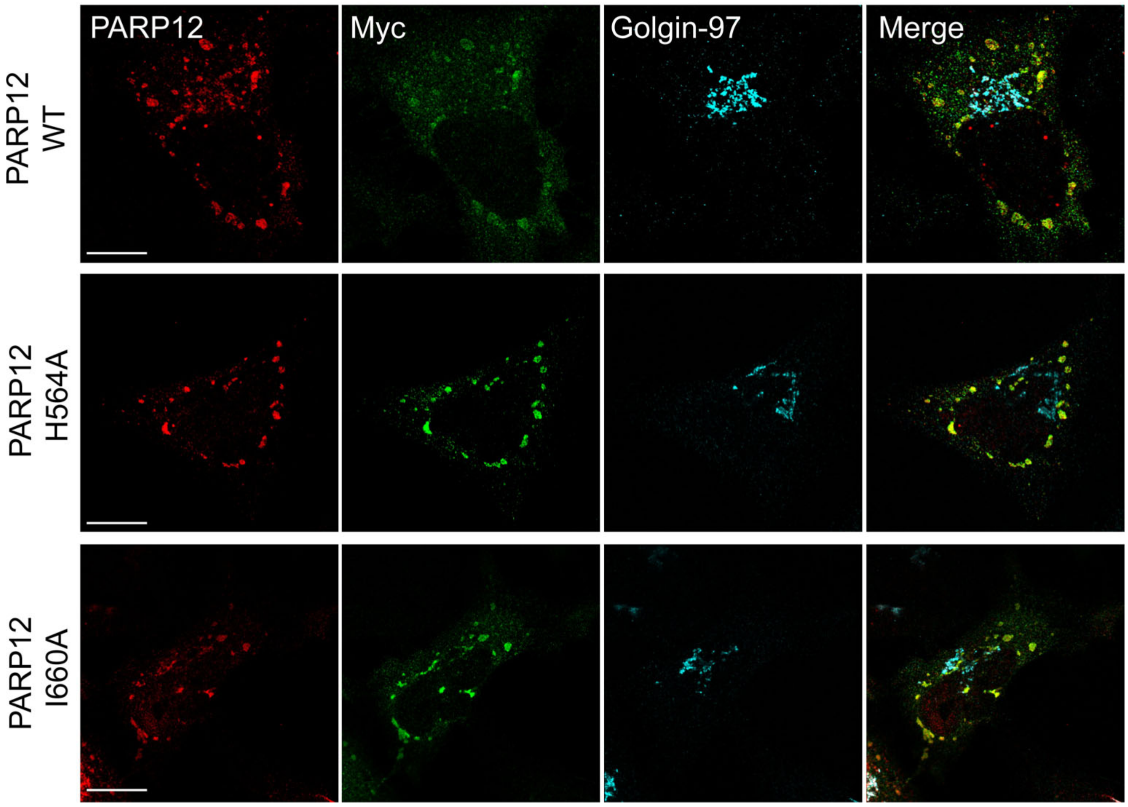

Supplementary Fig. 7. Analysis of PARP12 enzymatic activity and subcellular localization of PARP12 wild-type and catalytically inactive mutants. (a) Representative *in vitro* ADP-ribosylation assay of total membranes from HeLa cells transfected with empty vector or PARP12 wild-type His- or Myc-tagged (PARP12 His-WT or PARP12 Myc-WT) or different PARP12 Myc-tagged mutants (PARP12 Myc-H564A, PARP12 Myc-H564Q, PARP12 Myc-I660A) (Methods and Supplementary Table 1). The incorporated [^{32}P]ADP-ribose was detected by autoradiography (AR [^{32}P]) and the expression levels of PARP12 wild-type and mutants in the membrane fractions were monitored by Western blotting with anti-PARP12 antibody (WB: PARP12). Note that the different overexpression levels do not effect the conclusions of the two mutants acting as catalytically

inactive. Uncropped images of blots are shown in Supplementary Fig. 21. (**b**) Representative confocal microscopy images of HeLa cells transfected with empty vector or Myc-tagged PARP12 WT (PARP12 WT) or with the catalytically inactive mutants (PARP12 H564A, PARP12 I660A, as indicated) fixed and labeled with anti-PARP12 (red), anti-Myc (green) and anti-Golgin-97 (cyan) antibodies. Scale bars, 10 μm. The lack of PARP12 staining at the Golgi complex indicates that both the endogenous and overexpressed enzymes move to the stress granules.

Supplementary Fig. 8

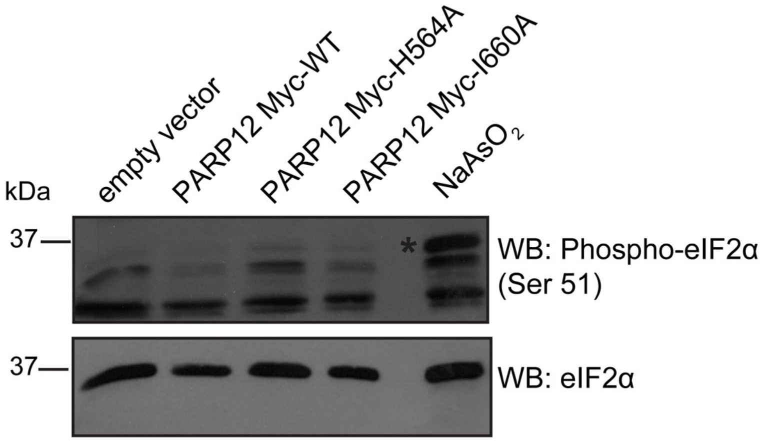

Supplementary Fig. 8. Immunodetection of phospho-eIF2α in HeLa cells expressing PARP12 wild-type and catalytically inactive mutants or treated with $NaAsO_2$. Western blotting with anti-phospho-eIF2α antibody to reveal the specific Ser51 phosphorylation of eIF2α [phospho-eIF2α (Ser51)] from total cell lysates of HeLa cells transfected with empty vector or Myc-tagged PARP12 (PARP12 Myc-WT) or PARP12 mutants (PARP12 Myc-H564A or PARP12 Myc-I660A, as indicated). Lysates from $NaAsO_2$-treated HeLa cells ($NaAsO_2$) were used as positive control of phospho-Ser51 eIF2α antibody-specific signal (black asterisk, top panel). The blot was then reprobed with anti-eIF2α antibody for total eIF2α protein (bottom panel). Molecular weight standards (kDa) are indicated on the left of each panel. Uncropped images of blots are shown in Supplementary Fig. 21.

Supplementary Fig. 9

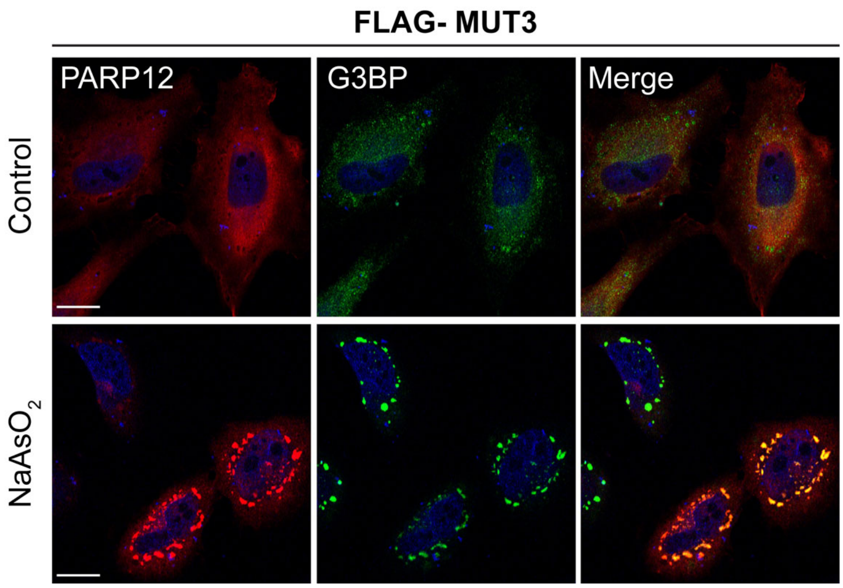

Supplementary Fig. 9. Localization of FLAG-tagged MUT3 of PARP12. Representative confocal microscopy images of HeLa cells expressing FLAG-MUT3 of PARP12, untreated (Control) or treated with $NaAsO_2$, fixed and labeled with anti-PARP12 (red) and anti-G3BP (green) antibodies. Scale bars, 10 μm.

Supplementary Fig. 10

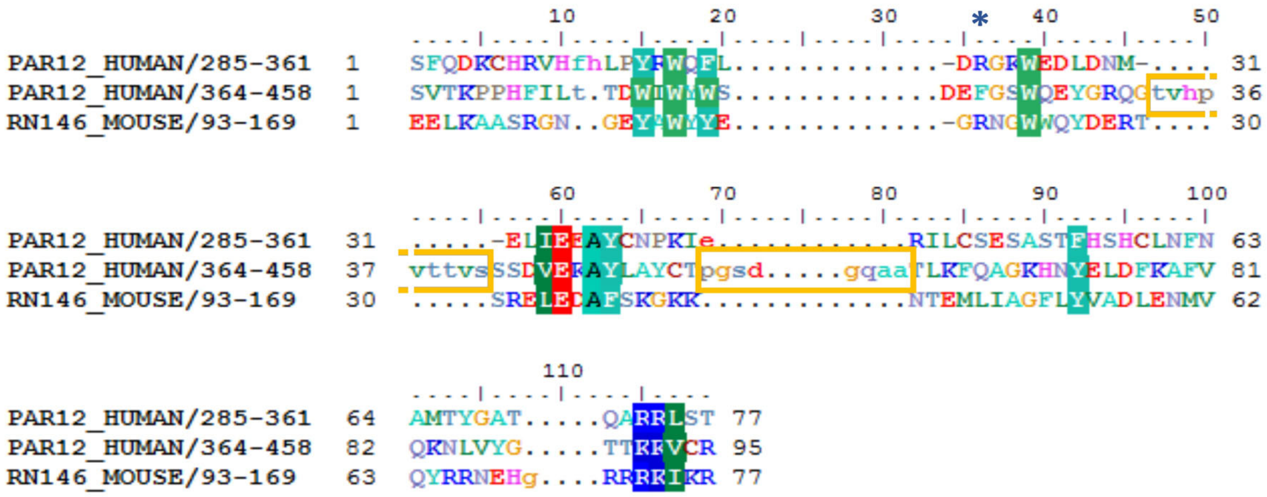

Supplementary Fig. 10. PARP12/RNF146 WWE domains sequence alignment taken from PROSITE class alignment PDOC50918 (http://prosite.expasy.org/PDOC50918). PARP12 WWE1 (residues 285-361), PARP12 WWE2 (residues 364-458) and RNF146 WWE (residues 93-169) sequence alignment. Identical/similar residues are highlighted with colored shades (similarity threshold at 90%). Asterisk indicates the alignment position of the key arginine responsible for one of the main anchoring interactions with the *iso*-ADP-ribose (this arginine is not present in the PARP12 WWE2 sequence). Orange boxes frame the insertions of nine and eight amino acid residues in PARP12 WWE2 sequence (these insertions would impact on the tree-dimensional structure of the putative *iso*-ADP-ribose binding domain). Lower-case characters represent insertion in the sequence respect to the motif alignment. Black bullets indicate the amino acid residues that define the signature pattern.

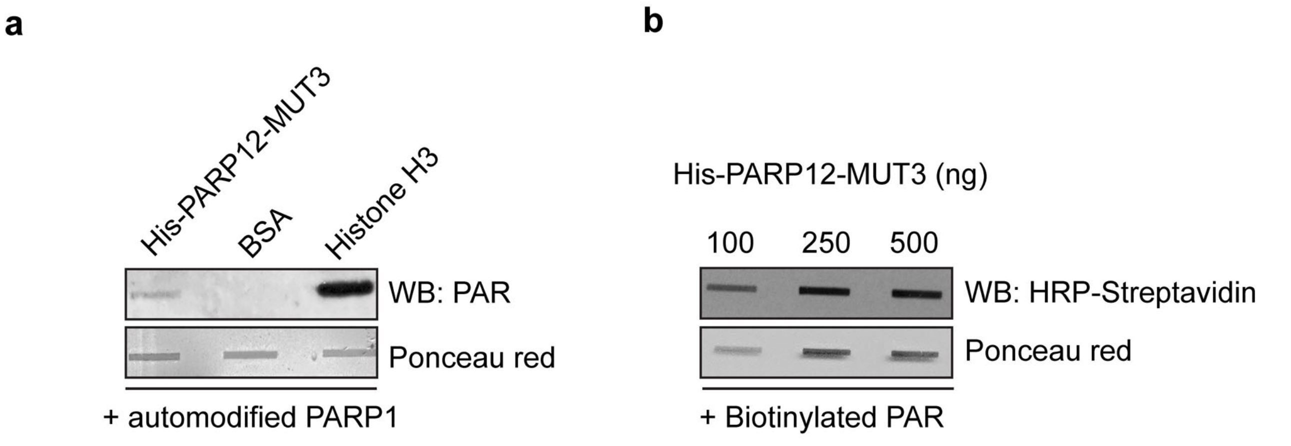

Supplementary Fig. 11. Binding of His-PARP12 MUT3 to PAR. **(a)** Far-Western blotting with purified His-PARP12-MUT3 immobilized on a nitrocellulose membrane and incubated with PARP1-bound PAR (+ automodified PARP1; see Methods) and revealed by Western Blotting using anti-PAR antibody (WB: PAR). BSA and Histone H3 proteins were used as internal negative and positive controls, respectively. Ponceau Red staining was used to visualize the amount of proteins. (**b**) Increasing amount of His-PARP12-MUT3 (100, 250 and 500 ng, as indicated) immobilized on a nitrocellulose membrane, incubated with free biotinylated-PAR (+ Biotinylated PAR) and revealed by Western Blotting using HRP-streptavidin (WB: HRP-Streptavidin). Ponceau Red staining was used to visualize the amount of proteins. Uncropped images of blots are shown in Supplementary Fig. 21.

Supplementary Fig. 12

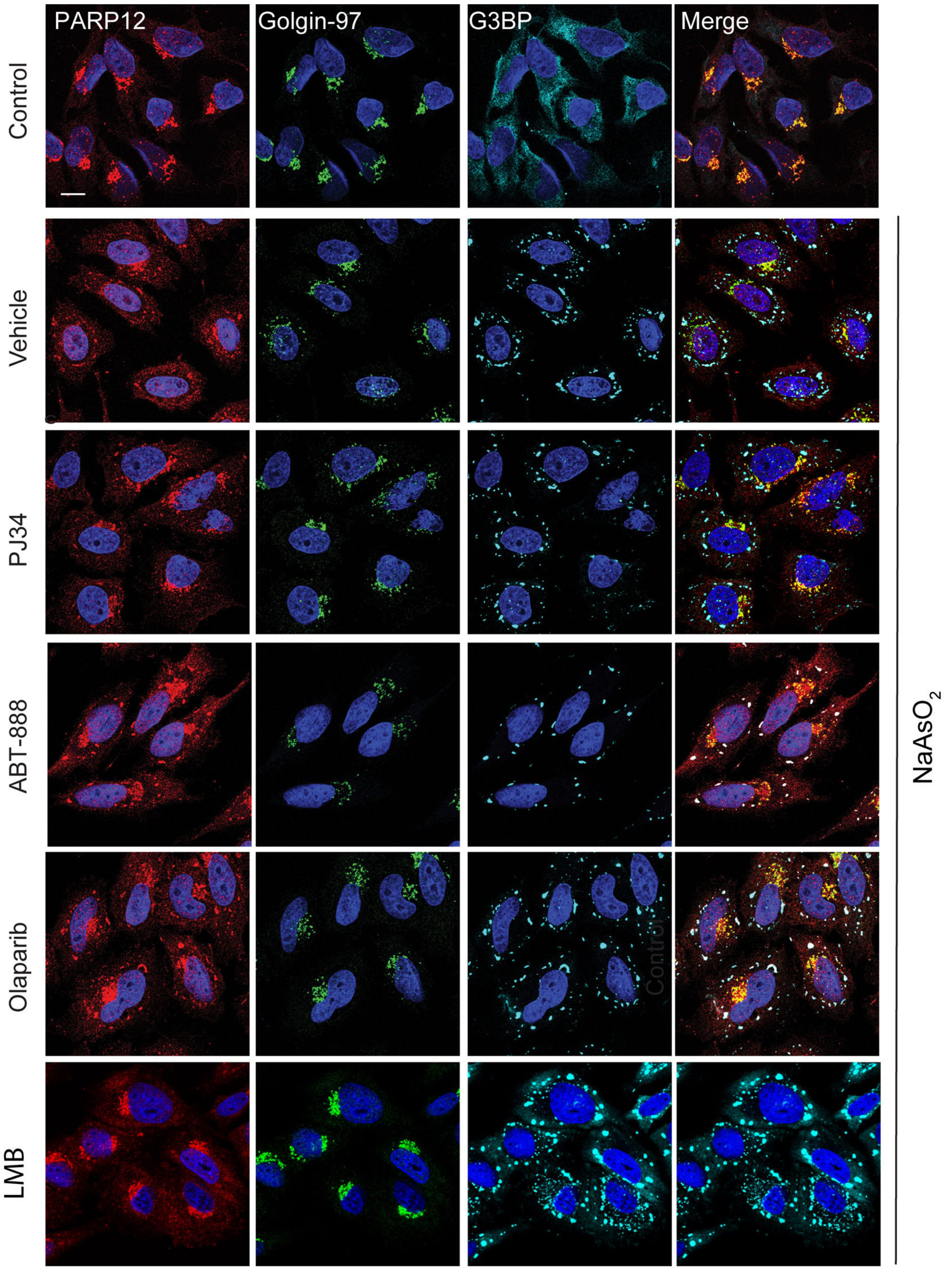

Supplementary Fig. 12. Inhibitory effects of PJ34, ABT-888, Olaparib and Leptomycin B on PARP12 translocation from the Golgi complex to stress granules upon oxidative stress. Representative confocal microscopy images of HeLa cells untreated (Control) or pre-treated for 16 h with PJ34 (30 μM), ABT-888 (45 μM), Olaparib (30 μM), Leptomycin B (LMB, 10 ng/ml) or DMSO (Vehicle) before exposure to 250 μM $NaAsO_2$ for a further 2 h (Methods). Cells were fixed and labeled with anti-PARP12 (red), anti-Golgin-97 (green) and anti-G3BP (cyan) antibodies. Scale bar, 10 μm.

Supplementary Fig. 13

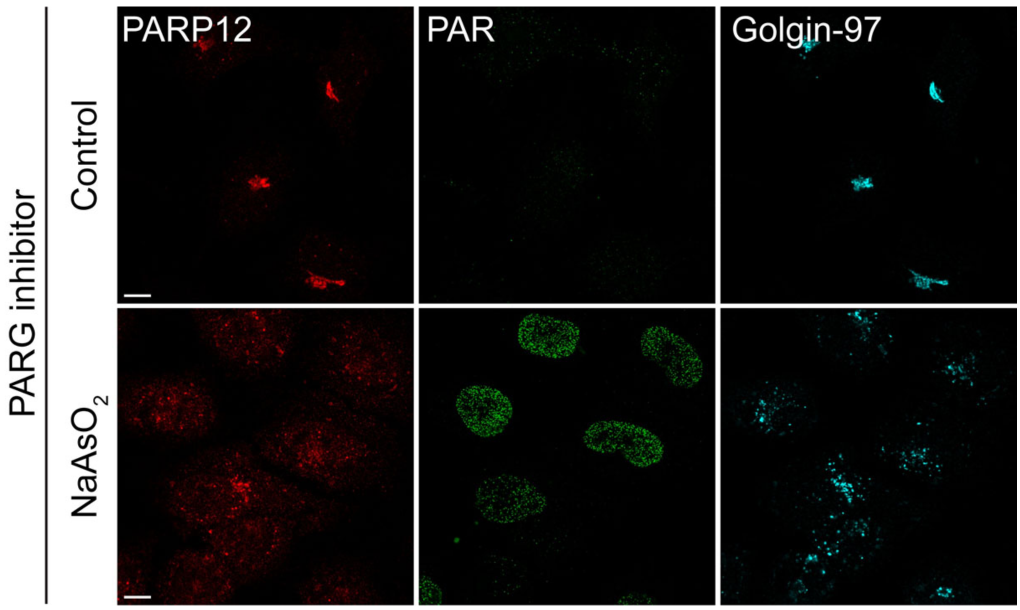

Supplementary Fig. 13. PARG inhibitor treatment does not affect PARP12 localization under oxidative stress. Representative confocal microscopy images of HeLa cells treated with PARG inhibitor (PDD00017238, 500 nM, 12 h) and then exposed to $NaAsO_2$ (250 μM), fixed and labeled with anti-PARP12 (red), anti-PAR (green) and anti-Golgin-97 (cyan) antibodies. See Methods for details. Scale bars, 10 μm.

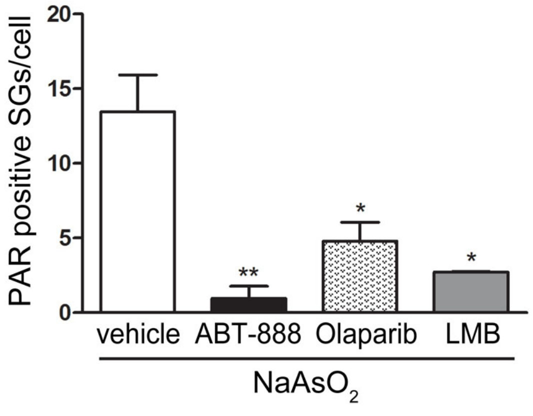

Supplementary Fig. 14. Inhibitory effects of ABT-888, Olaparib and Leptomycin B on PAR positive stress granules in presence of $NaAsO_2$. Quantification of PAR-positive stress granules (SGs) in HeLa cells pre-treated for 16 h with ABT-888 (45 μM), Olaparib (30 μM), DMSO (vehicle) or with the nuclear export inhibitor Leptomycin B (LMB, 10 ng/ml) and then exposed to 250 μM $NaAsO_2$ for a further 2 h (Methods). Cells were fixed and labeled with anti-PAR specific antibody. Data are mean ±SD from three independent experiments. Statistical significance was calculated by unpaired Student's t-test; *$p < 0.05$; **$p < 0.01$.

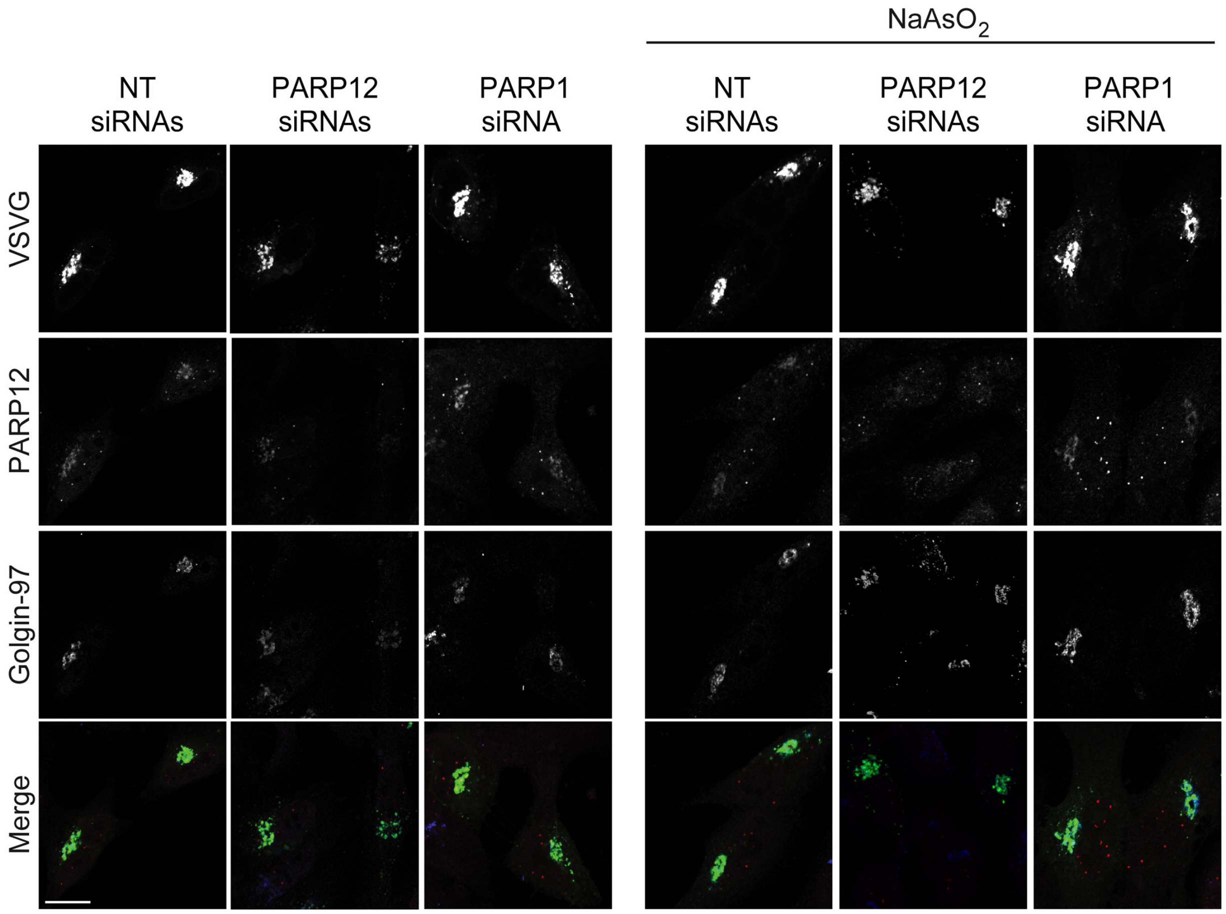

Supplementary Fig. 15. Oxidative stress during intracellular trafficking. Representative confocal microscopy images of VSVG transport in HeLa cells transfected with 150 nM non-targeting siRNAs (NT siRNAs) or 100 nM PARP12 siRNAs or 150 nM PARP1 siRNA before co-transfection for the last 16 h with VSVG-YFP (Methods). During the TGN-exit assay, after 90 min at 20 °C, the cells were untreated or treated with 250 μM $NaAsO_2$ ($NaAsO_2$) and incubated for a further 90 min at 20 °C. Cells were fixed after the 20 °C temperature-block and labeled with anti-PARP12 (red) and anti-Golgin-97 (blue) antibodies. Scale bar, 10 μm. See also Fig. 8 for trafficking effects after 120 min of the 32 °C temperature-block release and for efficiency of PARP1 and PARP12 interference.

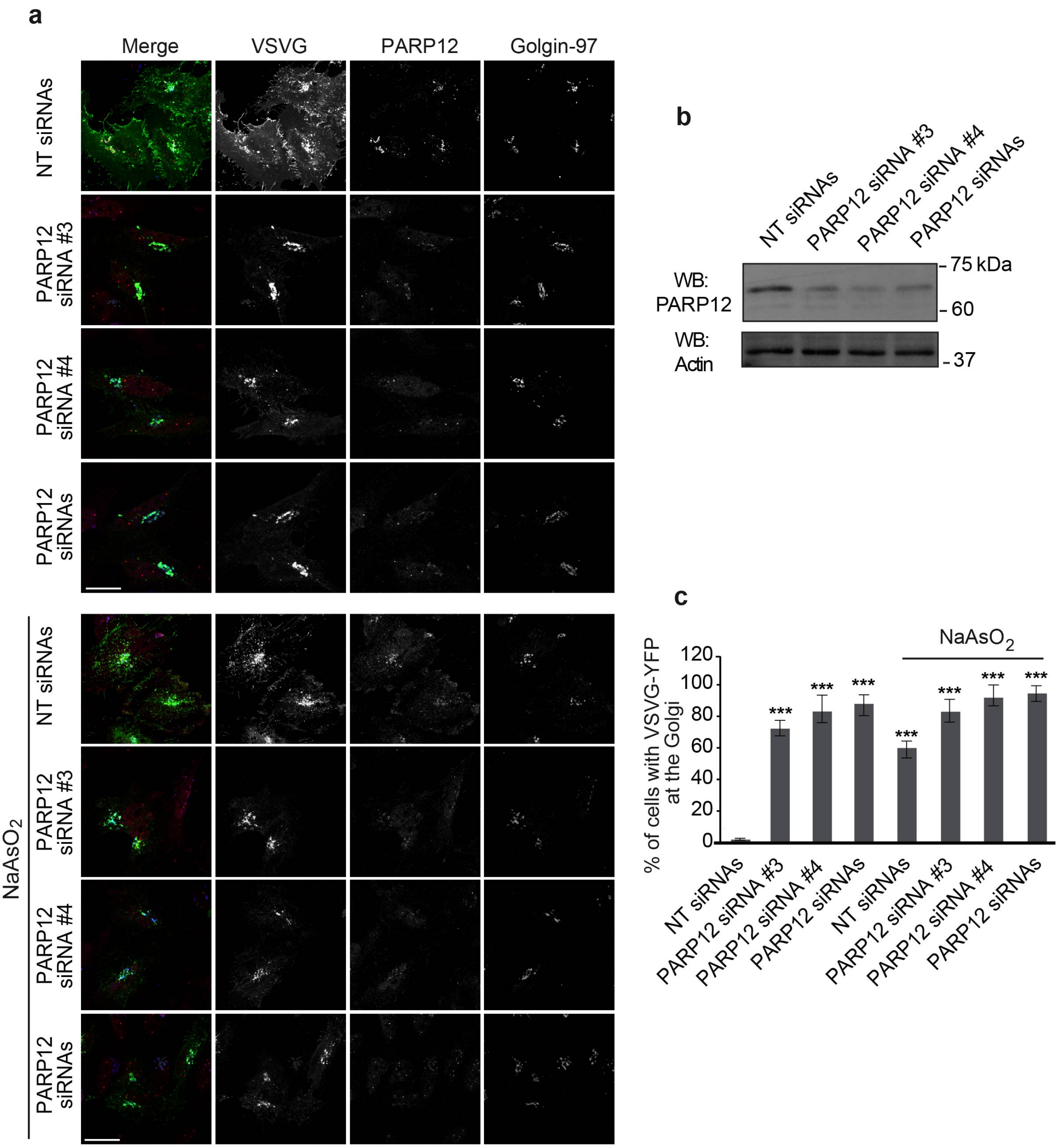

Supplementary Fig. 16. Effects of PARP12 depletion and oxidative stress during intracellular trafficking. (a) Representative confocal microscopy images of VSVG transport in HeLa cells transfected with 100 nM non-targeting siRNAs (NT siRNAs) or 100 nM PARP12 siRNA duplex #3 (PARP12 siRNA #3) or PARP12 siRNA duplex #4 (PARP12 siRNA #4) or PARP12 siRNAs pool (PARP12 siRNAs) before co-transfection for the last 16 h with VSVG-YFP (Methods). During the TGN-exit assay, after 90 min at 20 °C, the cells were untreated or treated with 250 μM $NaAsO_2$

($NaAsO_2$) and incubated for a further 90 min at 20 °C. Cells were fixed after 90 min of the 32 °C temperature-block release and labeled with anti-PARP12 (red) and anti-Golgin-97 (blue) antibodies. **(b)** Efficiency of interference by Western blotting of total cell lysates (25 μg/lane) with anti-PARP12 antibody (WB: PARP12). Actin is shown for the internal protein levels and molecular weight standards (kDa) are indicated on the right of the panel. Uncropped images of blots are shown in Supplementary Fig. 21. (**c**) Quantification of VSVG-YFP in the Golgi area after 90 min of the 32 °C temperature-block release in cells treated as in **a**. Data are mean ±SD from three independent experiments. Statistical significance was calculated by unpaired Student's t-test; *p* *** < 0.001 Scale bars, 10 μm.

Supplementary Fig. 17

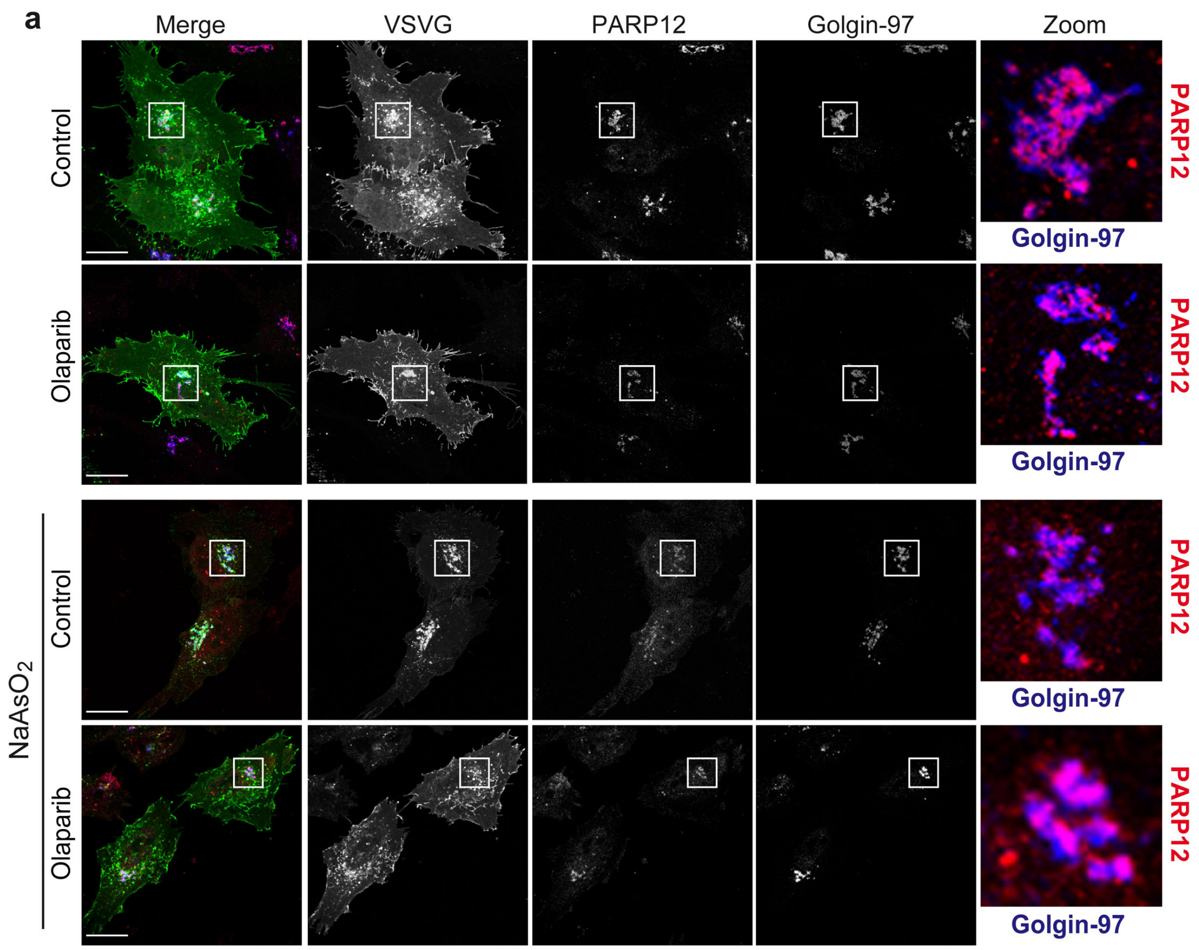

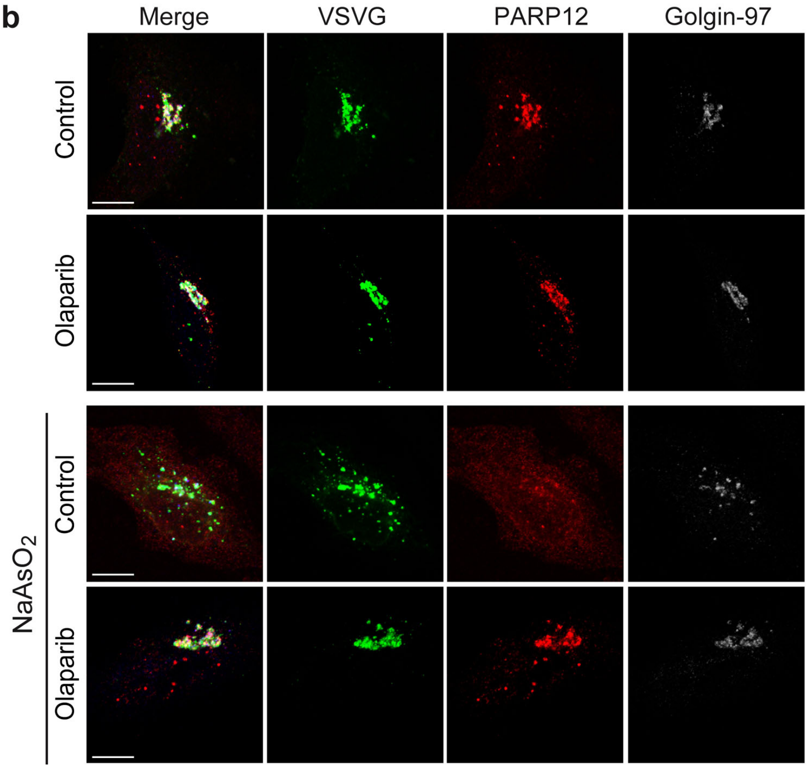

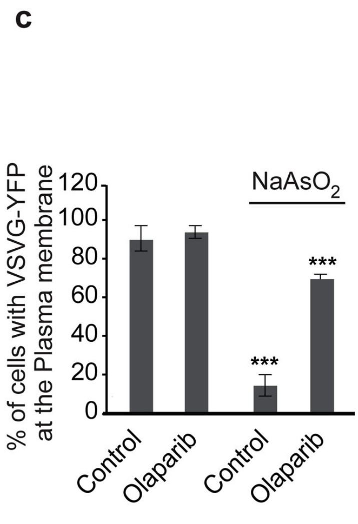

Supplementary Fig. 17. Effects of Olaparib treatment and oxidative stress during intracellular trafficking. (**a**) Representative confocal microscopy images of VSVG transport in HeLa cells transfected with VSVG-YFP. During the TGN-exit assay, the cells were untreated (Control) or treated with 100 μM Olaparib (Olaparib) at 20 °C for 3.5 h following a further 90 min incubation at 20 °C without or with 250 μM $NaAsO_2$ ($NaAsO_2$). Cells were then fixed after 90 min of the 32 °C temperature-block release and labeled with anti-PARP12 (red) and anti-Golgin-97 (blue) antibodies. Insets: magnification of Golgi area. (**b**) Representative confocal microscopy images of VSVG transport in HeLa cells treated as in **a,** and fixed after the 20 °C temperature-block and labeled with anti-PARP12 (red) and anti-Golgin-97 (blue) antibodies. (**c**) Quantification of VSVG-YFP in the Golgi area after 90 min of the 32 °C temperature-block release in cells treated as in **a**. Data are mean ±SD from three independent experiments. Statistical significance was calculated by unpaired Student's t-test; p *** < 0.001 Scale bars, 10 μm.

Supplementary Fig. 18

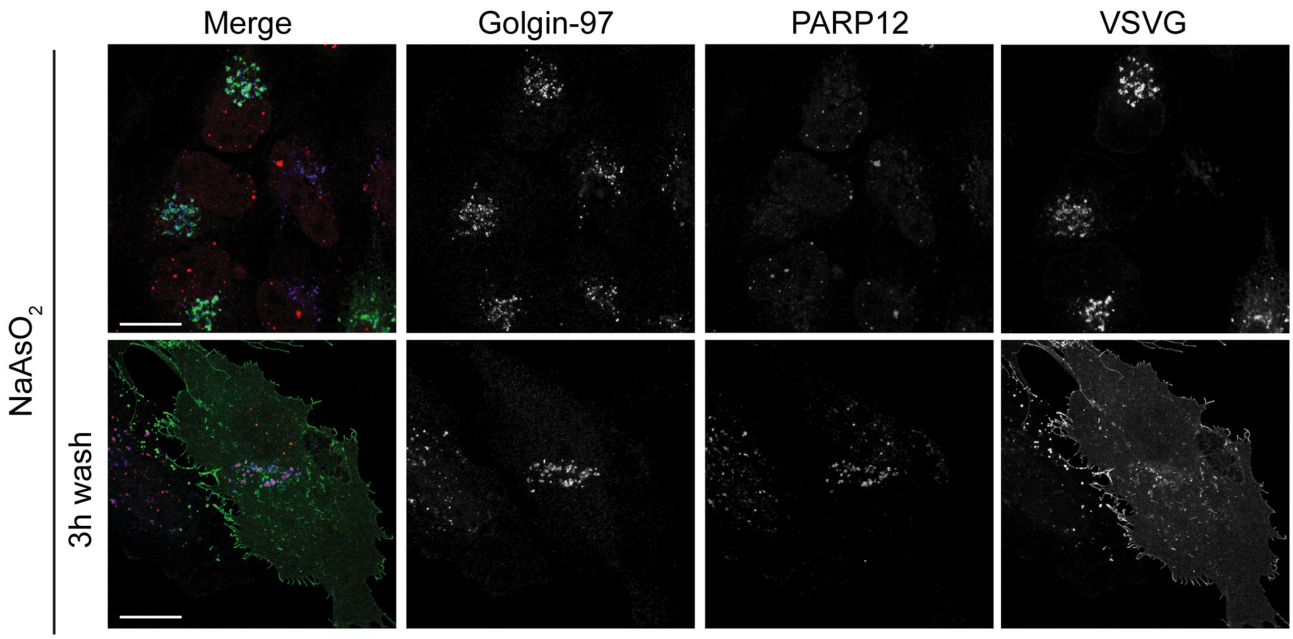

Supplementary Fig. 18. Oxidative stress impairs intracellular trafficking in a reversible manner. Representative confocal microscopy images of VSVG transport in cells treated with $NaAsO_2$ (see Methods for details). After 2 h of $NaAsO_2$ treatment ($NaAsO_2$), cells were fixed and labeled with anti-Golgin-97 (blue) and anti-PARP12 (red) antibodies. Alternatively, the drug was removed and cells fixed after 3 h from the drug wash-out (3 h wash). Scale bar, 10 μm.

Supplementary Fig. 19

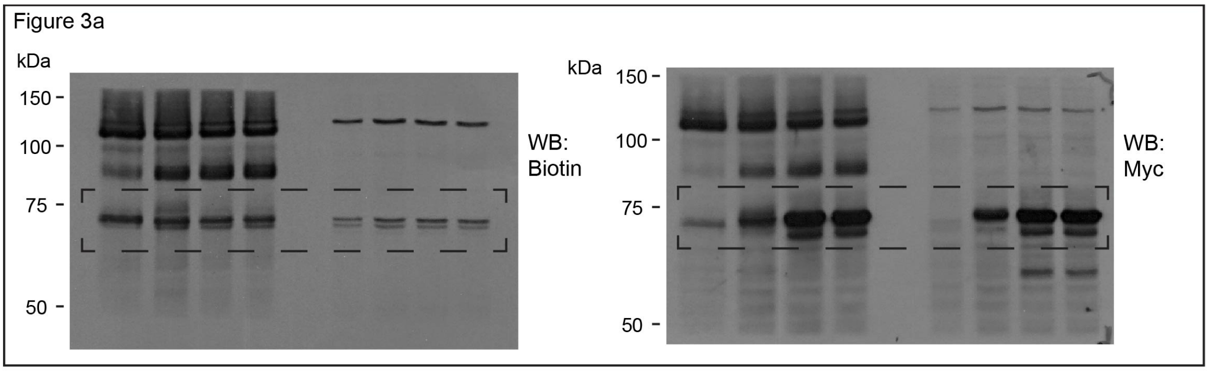

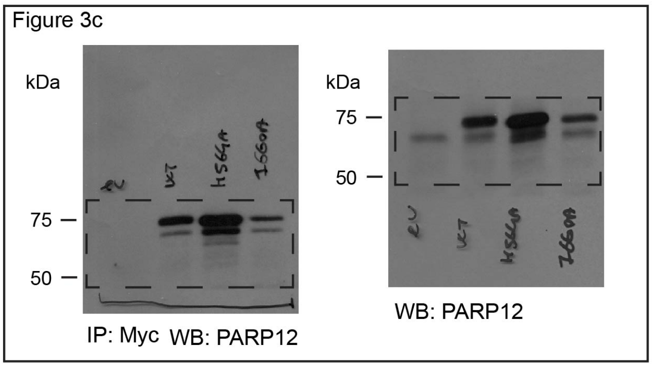

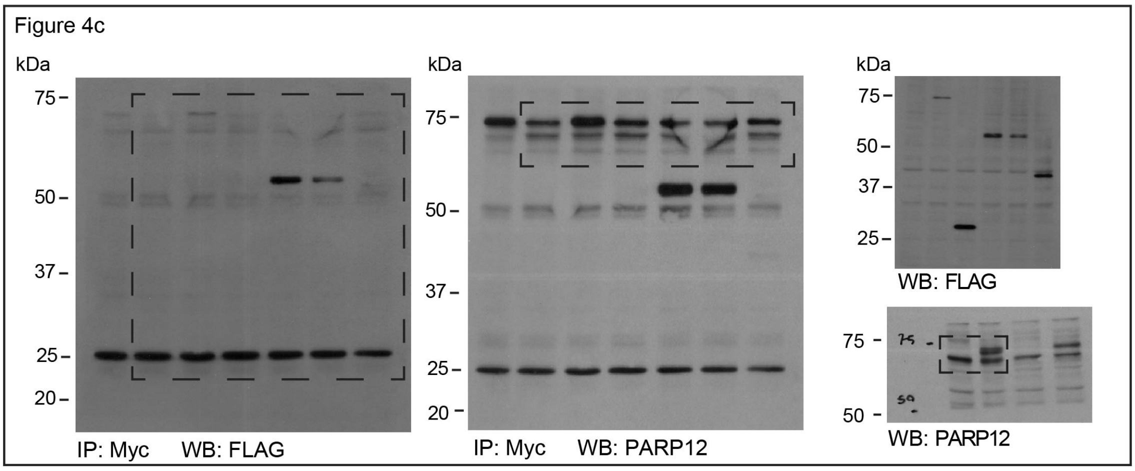

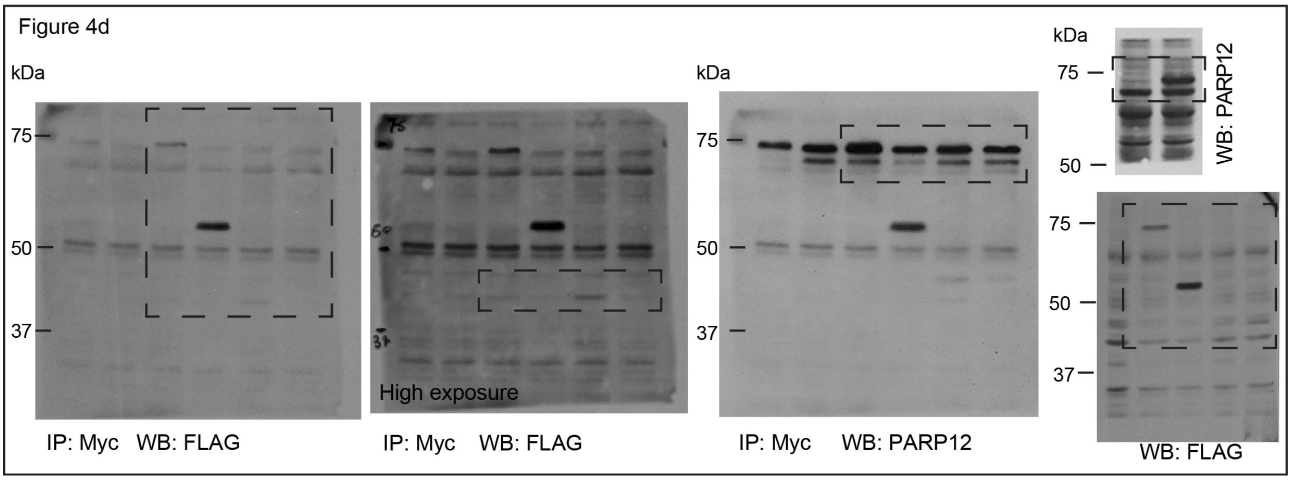

Supplementary Fig. 19. Full scan images of Western blotting data.

Supplementary Fig. 20

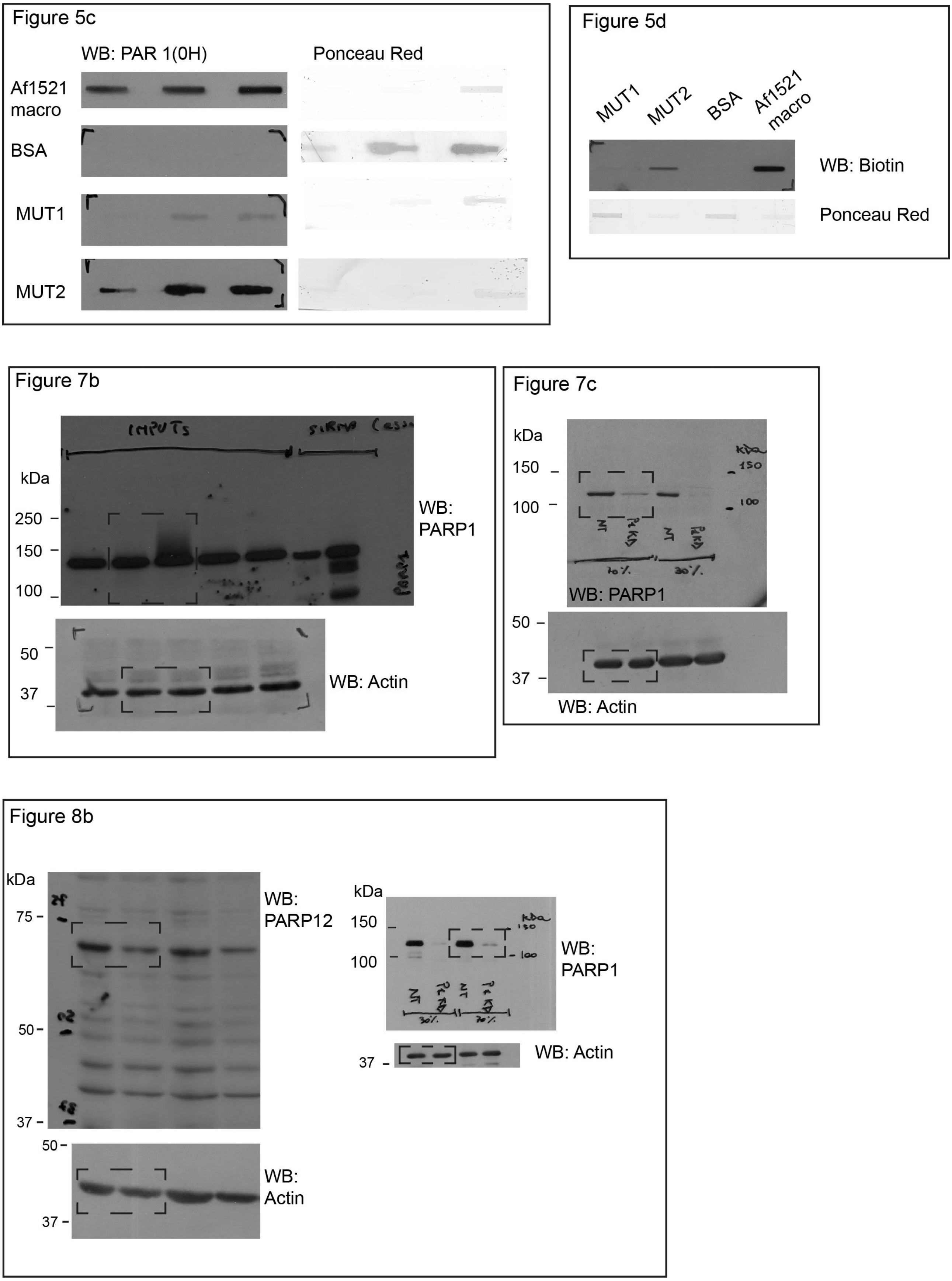

Supplementary Fig. 20. Full scan images of Western blotting and Ponceau staining data.

Supplementary Fig. 21

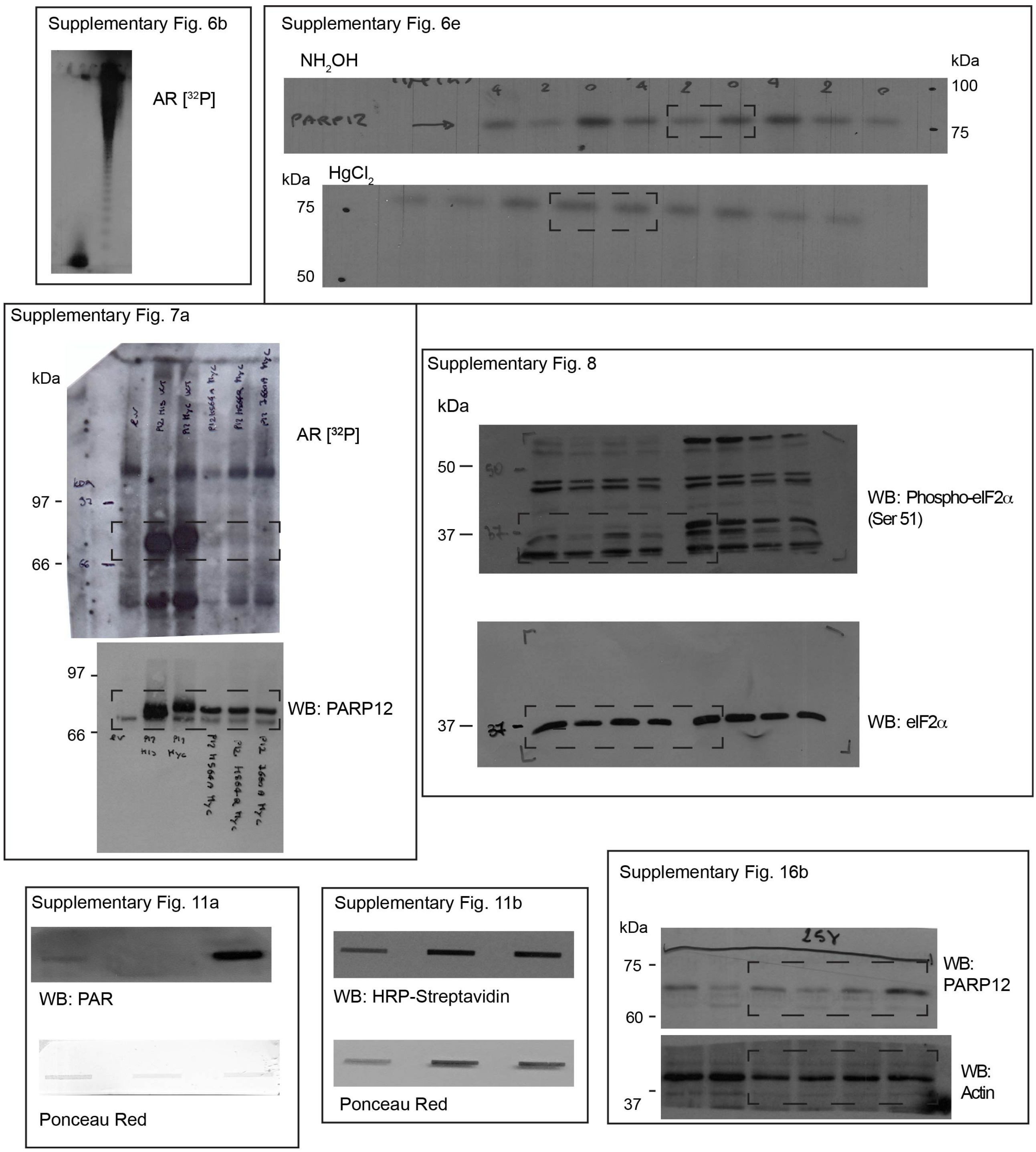

Supplementary Fig. 21. Full scan images of Western blotting and Ponceau staining data.
